# A gene expression atlas of a *bicoid*-depleted *Drosophila* embryo reveals early canalization of cell fate

**DOI:** 10.1242/dev.117796

**Published:** 2015-02-01

**Authors:** Max V. Staller, Charless C. Fowlkes, Meghan D. J. Bragdon, Zeba Wunderlich, Javier Estrada, Angela H. DePace

**Affiliations:** 1 Department of Systems Biology, Harvard Medical School, Boston, MA 02115, USA; 2 Department of Computer Science, University of California Irvine, Irvine, CA 92697, USA

**Keywords:** Canalization, *Drosophila*, Bicoid, Even-skipped, Transcriptional network, Gene expression atlas

## Abstract

In developing embryos, gene regulatory networks drive cells towards discrete terminal fates, a process called canalization. We studied the behavior of the anterior-posterior segmentation network in *Drosophila melanogaster* embryos by depleting a key maternal input, *bicoid* (*bcd*), and measuring gene expression patterns of the network at cellular resolution. This method results in a gene expression atlas containing the levels of mRNA or protein expression of 13 core patterning genes over six time points for every cell of the blastoderm embryo. This is the first cellular resolution dataset of a genetically perturbed *Drosophila* embryo that captures all cells in 3D. We describe the technical developments required to build this atlas and how the method can be employed and extended by others. We also analyze this novel dataset to characterize the degree and timing of cell fate canalization in the segmentation network. We find that in two layers of this gene regulatory network, following depletion of *bcd*, individual cells rapidly canalize towards normal cell fates. This result supports the hypothesis that the segmentation network directly canalizes cell fate, rather than an alternative hypothesis whereby cells are initially mis-specified and later eliminated by apoptosis. Our gene expression atlas provides a high resolution picture of a classic perturbation and will enable further computational modeling of canalization and gene regulation in this transcriptional network.

## Abstract (precis)

**Summary**: *Drosophila bicoid* mutant embryos show severe patterning phenotypes, but individual cells retain wild-type fates - as judged by expression of key patterning genes at single-cell resolution.

## INTRODUCTION

Specialization of cell fate underlies the diversity of metazoan form and function. Cell fates are specified robustly and precisely by gene regulatory networks that pattern embryos (Davidson, 2006). A key challenge in development is to understand how gene regulatory networks specify cell fates. The *Drosophila melanogaster* blastoderm embryo is a premier system for coupling computational models and quantitative experimental data to test hypotheses about the design of developmental networks (Reinitz and Sharp, 1995; Jaeger et al., 2004a,b; Poustelnikova et al., 2004; Hengenius et al., 2011; Papetsenko and Levine, 2011). Anterior-posterior patterning of the embryo is controlled by the well-characterized segmentation network (Lawrence, 1992; St Johnston and Nüsslein-Volhard, 1992; Jaeger et al., 2012). Computational models of this network have tested the sufficiency of known connections, proposed new connections and tested network-level properties (reviewed by Wunderlich and DePace, 2011; Jaeger et al., 2012).

Gene expression atlases enable the study of network properties. These atlases combine measurements of mRNA or protein expression from many individual embryos into an average embryo; the resulting data are in a computationally amenable format with high resolution in space and time. The first such dataset, the FlyEx database (Poustelnikova et al., 2004; Pisarev et al., 2009), was a 1D anterior-posterior atlas that triggered a renaissance in computational modeling of fly patterning and transcriptional control (Jaeger et al., 2004b; Janssens et al., 2006; Segal et al., 2008; He et al., 2010). The 3D atlas built by the Berkeley Drosophila Transcription Network Project (BDTNP) (Keränen et al., 2006; Luengo Hendriks et al., 2006, 2007; Fowlkes et al., 2008) enabled similar approaches in every cell of the embryo (Bieler et al., 2011; Hengenius et al., 2011; Umulis and Othmer, 2012; Ilsley et al., 2013; Samee and Sinha, 2013). Extending 3D atlas building methods to other species enabled comparative analysis of transcriptional circuits (Fowlkes et al., 2011; Wunderlich et al., 2012).

The existing wild-type atlases allow for fitting and cross-validation of computational models, but a gold standard for testing computational models is whether they can predict how a system behaves under genetic perturbation. This strategy has been difficult to apply because of limited quantitative data for mutant embryos. It is common to simulate the effect of a mutation and qualitatively compare the computational model predictions to published images in the literature (Papatsenko and Levine, 2008; Ilsley et al., 2013). However, it is difficult to accurately simulate mutant embryos because both direct and indirect effects are prevalent. To validate computational models, it is clearly preferable to collect direct quantitative measurements of entire gene regulatory networks in mutant embryos (Kozlov et al., 2012; Janssens et al., 2013; Surkova et al., 2013).

Here, we present a 3D gene expression atlas of a *Drosophila* blastoderm embryo depleted of the maternal transcription factor *bicoid* (*bcd*). To build this atlas, we overcame two technical challenges: first, collecting enough embryos for high throughput imaging; and second, controlling phenotypic variability. To solve the first problem, we used the maternal Gal4 shRNA system (Staller et al., 2013). shRNA depletion is genetically dominant, avoids labor-intensive sorting of mutant females, and will enable biochemical analysis in future work. The second problem, phenotypic variation, is shared by shRNA depletion and mutant alleles (Waddington, 1942). We reduced variability both experimentally and by curating our images so that the resulting atlas represents the most common phenotypic class. Both of these technical developments will be applicable to building gene expression atlases of additional genetic perturbations in the future.

Our goal in building this atlas was not to investigate *bcd* behavior per se, but to determine how individual cells respond to a dramatic perturbation of the segmentation network. Bcd protein activates head cell fates and represses posterior cell fates (Lawrence, 1992). Deleting *bcd* leads to duplication of posterior structures in the anterior, a strong perturbation of cell fate specification. More subtle perturbations, such as variations in Bcd levels, have been useful for computational modeling of segmentation network behavior (Manu et al., 2009a,b; Gursky et al., 2011; Liu et al., 2013). Our *bcd*-depleted gene expression atlas combines data for 13 key segmentation genes and seven reporters for enhancers that respond to these genes into a single morphological framework for six time points in blastoderm embryos. This atlas captures the direct and indirect effects of *bcd* depletion on the segmentation network for every cell of the embryo.

We used our atlas of the *bcd*-depleted embryo to investigate canalization of cell fate in individual cells. In his 1942 paper, Conrad Waddington used genetic and embryological evidence to support the idea that development canalizes cell fate (Waddington, 1942, 1957). Each of these lines of evidence has developed into a different modern definition of canalization*.* First, wild-type individuals are phenotypically highly reproducible whereas mutant populations are more variable; this genetic evidence leads to one definition, that developmental systems buffer genetic and environmental perturbations to create stereotyped individuals, reducing phenotypic variability over time. Second, differentiated cells and tissues are distinct; this embryological evidence leads to the second definition, that developmental systems create discrete cell fates, avoiding hybrids. We focused primarily on the second definition of canalization: developmental systems create discrete cell fates.

To examine canalization, we used cellular gene expression patterns as a proxy for cell fate (Waddington, 1957). We defined gene expression patterns (and thus cell fate) as combinations of key transcription factors. This strategy allowed us to ask whether any new cell fates emerged in the *bcd*-depleted embryo. We examined two layers of the segmentation network, the gap genes and the pair-rule genes. We found that all gap gene cell fates present in the *bcd*-depleted embryo were also present in wild type. For the pair-rule genes, we observed that extensive early overlap of *even-skipped* (*eve*) and *fushi-tarazu* (*ftz*) mRNA patterns in *bcd*-depleted embryos resolved into mutually exclusive domains. These two results support the hypothesis that canalization is directly mediated by the segmentation network and occurs during the blastoderm stage. The techniques we describe can be readily applied to other genetic perturbations, and the gene expression atlas of a *bcd* depleted embryo we present will be a useful resource for computational analysis and modeling of gene regulation in *Drosophila* blastoderm embryos.

## RESULTS

### Maternal Gal4 shRNA knockdown of *bcd* phenocopies mutant alleles

**Fig. 1.**
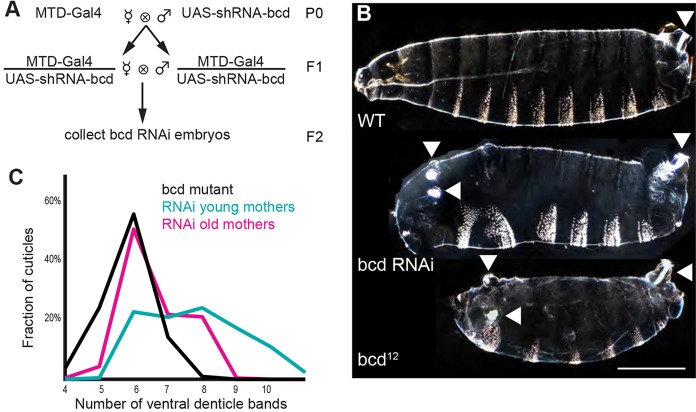
**Gal4-driven shRNA against *bcd* in the female germline phenocopies *bcd*-mutant alleles.** (A) Crossing scheme for generating *bcd* RNAi embryos (see Materials and Methods). (B) Top: dark-field image of a wild-type larval cuticle. The white patches of bristles on each segment are the ventral denticle bands. Middle: *bcd* RNAi cuticle. Bottom: *bcd* mutant cuticle. The *bcd* RNAi embryo has several key features of a classic *bcd* mutant, including the absence of all head and thoracic structures and the unextended ectopic filzkörper (anterior arrowheads; posterior arrowheads indicate the endogenous extended filzkörper). All cuticles are oriented with anterior towards the left and ventral towards the bottom. Scale bar: 200 μm. (C) The strength of knockdown increases and the phenotypic variability decreases as the *MTD-Gal4/UAS-shRNA-bcd* mothers age. Mutant, *n*=216; old mothers, *n*=253, day 15; young mothers, *n*=217, day 3. Coefficients of variation: mutant=0.127; old mothers=0.136; young mothers=0.180.

To collect the large quantities of embryos necessary to build a gene expression atlas, we used the ‘maternal Gal4 shRNA’ system to deplete *bcd* mRNA in the female germ line (Ni et al., 2011; Staller et al., 2013). shRNAs are genetically dominant, a feature that ensures all embryos are affected while avoiding labor-intensive sorting of mutant females. The fly husbandry is simple and scalable, enabling biochemical and functional genomic analysis. The technique is extendable to other genes, can be more consistent than classic mutant alleles and is inducible in specific tissues, an advantage over CRISPR-Cas9 genome editing (Ren et al., 2013). We crossed *maternal triple driver Gal4* (*MTD*-*Gal4*) females with *UAS-shRNA-bcd* males and collected embryos laid by *MTD-Gal4/UAS-shRNA-bcd* females (Fig. 1A).

Mutant and shRNA embryos exhibited similar phenotypes (Fig. 1B) and phenotypic variability that needed to be controlled prior to building a gene expression atlas (Fig. 1C; supplementary material Fig. S1). We quantified phenotypic variability by counting the number of ventral denticle bands on each cuticle. Embryos laid by *MTD-Gal4/UAS-shRNA-bcd* females (*bcd* RNAi embryos) had a distribution of phenotypes that overlapped the distribution of embryos laid by *bcd* mutant females (*bcd* mutant embryos) (Frohnhöfer and Nüsslein-Volhard, 1986). The primary determinant of phenotypic strength and variability in *bcd* RNAi embryos was the age of the mothers: older mothers laid embryos with stronger and less variable phenotypes (Fig. 1C). This improvement may stem from a slowing of oogenesis in older females, permitting the shRNAs more time to deplete targets (Ni et al., 2011). To balance reducing variability against declining fecundity, we collected embryos after aging the flies in cages for at least 11 days, at which point >90% of embryos passed our threshold for a strong *bcd* phenotype: eight or fewer denticle bands (all abdominal) and ectopic tail structures (supplementary material Fig. S2A).

Cuticle preparations provide a fast and easy way to identify sources of variability. We tested the effect of temperature, shRNA sequence, maternal driver, paternal genotype and the number of *UAS-shRNA-bcd* transgenes in each embryo, but none contributed strongly to phenotypic variability (supplementary material Fig. S2C,D). The absence of any paternal or zygotic effects enabled introduction of enhancer *lacZ* reporters into the atlas (see Materials and Methods; supplementary material Table S1).

### Building a gene expression atlas of a *bcd*-depleted embryo

To build a gene expression atlas, many individually stained embryos are registered together using a common gene expression pattern (also known as a fiduciary marker) (Fowlkes et al., 2008). Registration requires a template embryo that captures both average embryo morphology (cell number and cell density) and the expression pattern of the fiduciary marker. Because *bcd* RNAi embryos differ in morphology and fiduciary marker expression (supplementary material Fig. S3), we built a new template. We built our *bcd* RNAi registration template using 249 embryos stained only for *ftz* mRNA. In principle, many genes could serve as a fiduciary marker. In wild-type embryos either *eve* or *ftz* was used (Fowlkes et al., 2008, 2011). We chose *ftz* because the probe is very reliable. At late time points, some embryos expressed an extra *ftz* stripe; these individuals were excluded from the dataset.

### Characteristics of the *bcd* RNAi gene expression atlas

**Fig. 2.**
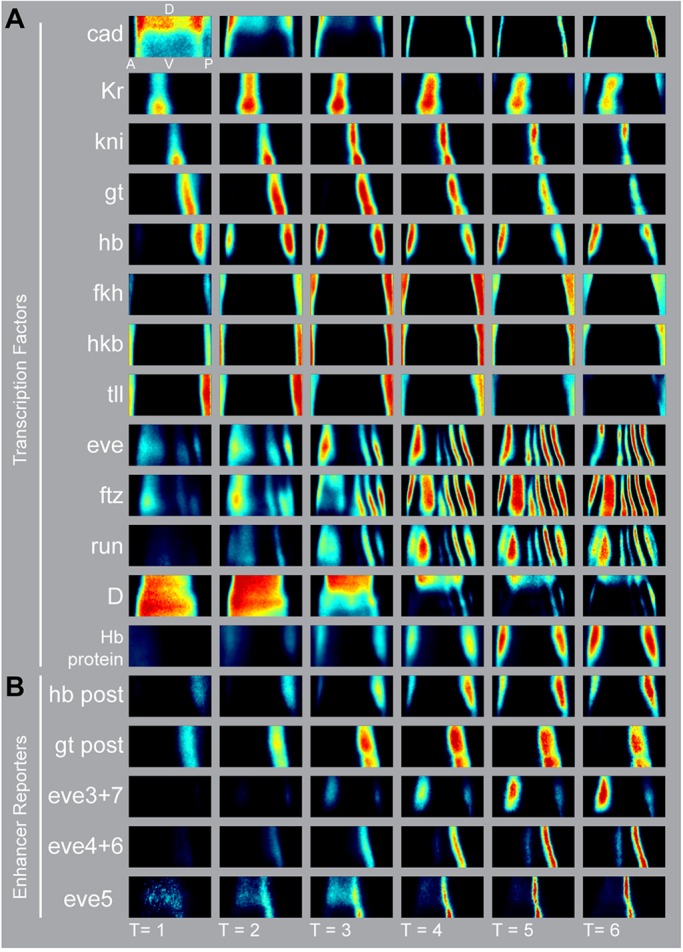
**The gene expression atlas of a *bcd*-depleted embryo highlights the expansion of trunk patterns, duplication of posterior patterns and loss of anterior patterns.** (A) Heat maps for mRNA expression patterns of 13 genes in the *bcd* RNAi atlas. Unrolled lateral views of half of the embryo are shown. Anterior is left; dorsal is top. Relative mRNA levels scale from no expression (black) to peak expression (red). We also collected Hb protein data. We partition the data into six ∼10 min cohorts that span all of stage 5 using a morphological marker (see Materials and Methods). (B) Heat maps for mRNA expression patterns of five reporter constructs included in the atlas. A, anterior; P, posterior; D, dorsal; V, ventral.

The *bcd* RNAi atlas includes 1817 embryos with mRNA stains for *caudal* (*cad*), *Krüppel* (*Kr*), *knirps* (*kni*), *giant* (*gt*), *hunchback* (*hb*), *fork head* (*fkh*), *huckebein* (*hkb*), *tailless* (*tll*), *Dichaete* (*D*), *runt* (*run*), *hairy* (*h*), *even-skipped* (*eve*) and *fushi-tarazu* (*ftz*) (Fig. 2; embryos per gene in supplementary material Table S2)*.* In addition, we measured seven *lacZ* reporter constructs containing the following enhancers: *hb posterior*,* gt posterior*,* eve stripe3+7*,* eve stripe5*,** two *eve stripe4+6* enhancers, and whole locus *eve* reporter (gift from Miki Fujioka) (supplementary material Table S1). We also collected embryos carrying reporters for the *eve stripe1*,* eve stripe2*,** and *eve late seven stripe* enhancers, but these sequences drove very little expression in the blastoderm. Finally, we collected protein data for Hb, for which there is a large difference in the mRNA and protein patterns in both WT and *bcd* mutants (Fig. 2, supplementary material Fig. S4). In WT embryos, anterior Hb protein arises from translational regulation of maternal mRNA and *bcd* activated zygotic mRNA. In *bcd* RNAi embryos, in the anterior there is a broad maternally controlled pattern and single zygotic stripe, a duplication of the posterior stripe (Tautz, 1988; Hülskamp et al., 1989; Irish et al., 1989; Struhl et al., 1989). All gene expression patterns agree with published images (Frasch and Levine, 1987; Nüsslein-Volhard et al., 1987; Tautz, 1988; Hooper et al., 1989; Struhl et al., 1989; Hülskamp et al., 1990; Kraut and Levine, 1991a; Rivera-Pomar et al., 1995), but our high temporal and spatial resolution atlas reveals dynamics that were not always captured by published images.

**Fig. 3.**
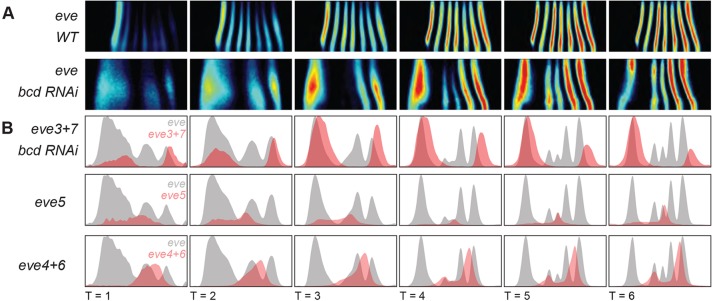
**Enhancer reporter constructs identify the *eve* stripes in *bcd* RNAi embryos.** (A) Heat maps for *eve* mRNA patterns in the wild-type and *bcd* RNAi gene expression atlases. Unrolled lateral views of half of the embryo are shown. Anterior is left; dorsal is up. mRNA expression scales from no expression (black) to peak expression (red). (B) Line traces of the endogenous *eve* pattern (gray) and the reporter (red) show anterior-posterior position on the *x*-axis and expression level on the *y*-axis for a single strip along the lateral side of the embryo. Because of the way the atlases are constructed, absolute mRNA expression levels cannot be compared between genotypes (see Wunderlich et al., 2014). Here, the levels of the reporter line traces have been manually scaled to match the corresponding endogenous stripe peak to highlight differences in the position of expression.

In *bcd* RNAi embryos, there is more variability in the pair-rule gene expression patterns than in the gap gene expression patterns. In 22/98 of *bcd* RNAi embryos, the anterior *eve* stripe split at T=5 and T=6. These embryos were excluded from the atlas. In embryos with a single anterior stripe, the position and width of this stripe varied more than the other stripes (supplementary material Fig. S5). Aside from the anterior stipe, the coefficients of variation (c.v.) of *eve* stripe widths were comparable with the c.v. of the gap gene widths, indicating that in this region both layers of the network had similar embryo-to-embryo variability (supplementary material Fig. S6). The boundaries of both the reporters and endogenous *eve* stripes refined later in *bcd* RNAi than in wild type (Figs 2 and 3, supplementary material Fig. S5).

The *bcd* RNAi gene expression atlas is of similar quality to the wild-type atlas. The standard deviation of each gene averaged over all cells and all time points is smaller in the *bcd* RNAi atlas than in the wild-type atlas for 10 out of 13 genes (supplementary material Table S3). Second, for all but a few genes, background expression levels in cells with low expression levels (OFF cells) are lower in *bcd* RNAi, as shown in the histogram of expression levels (see Fig. 5A). The atlas is freely available on FigShare at http://figshare.com/articles/A_gene_expression_atlas_of_a_bicoid_depleted_ Drosophila_embryo/1270915  (http://dx.doi.org/10.6084/m9.figshare.1270915).

### Identifying the perturbed *eve* stripes in *bcd* RNAi embryos

To correspond the five *eve* stripes in *bcd* RNAi embryos with their wild-type counterparts, we introduced *eve* enhancer reporter constructs into the *bcd* RNAi embryo (Figs 2 and 3). The *eve* locus contains five enhancers that together drive seven stripes (Goto et al., 1989; Small et al., 1991, 1996; Fujioka et al., 1999). To our knowledge, the stripe 4+6 and stripe 5 enhancer reporter constructs have not previously been examined in *bcd* mutant embryos. Consistent with the literature (Small et al., 1996), we found that the five *eve* stripes in *bcd* RNAi embryos correspond to *eve* stripes 3+7, *eve* stripes 4+6 and *eve* stripe 5 (Fig. 3).

### The gap gene expression patterns expand asymmetrically

**Fig. 4.**
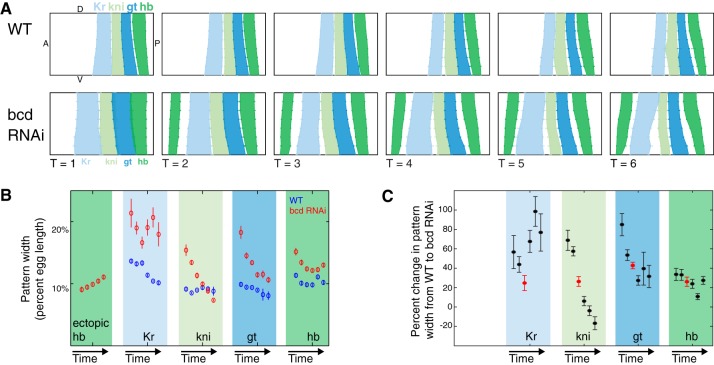
**The gap gene expression patterns in the trunk expand by different amounts in *bcd* RNAi embryos.** (A) The gap gene expression patterns in the trunk in wild-type and *bcd* RNAi gene expression atlases plotted on an unrolled lateral view of half the embryo. The pattern boundaries were calculated by finding the inflection point of lateral strips in individual embryos using the BDNTP PointCloud Toolbox (see Materials and Methods). Error bars indicate s.e.m. (B) The widths of each gap gene expression domain change over time in wild type (blue circles) and *bcd* RNAi (red circles). For each gene, the width of the pattern, calculated from a lateral strip, is plotted over six time points. The patterns narrow over time in both genotypes, but more quickly in *bcd* RNAi. Pattern widths plotted as percent egg length (EL). Distance between nuclei is ∼1% EL. Error bars indicate s.e.m. (C) The percentage change in gap gene expression domain widths between wild type and *bcd* RNAi, calculated for each time point from B. All time points are ordered left from to right in each column (black circles); time point 3 is indicated in red.

A prominent feature of the *bcd* RNAi embryo is the asymmetric expansion of the gap gene expression patterns. In wild type, the anterior boundary of *Kr* begins at 44% egg length (from the anterior) and the *Kr*, *kni*, *gt*, *hb* and *tll* patterns fill the remaining 56% of the embryo. In *bcd* RNAi, the anterior boundary of *Kr* shifts to begin at 27% egg length, and the gap gene domains expand to fill 73% of the embryo (Fig. 4). Although individual pattern shifts have been noted in the past (Struhl et al., 1989; Hülskamp et al., 1990; Kraut and Levine, 1991a; Rivera-Pomar et al., 1995), our measurements revealed that each pattern expanded by a different amount and had unique dynamics (Fig. 4B). The asymmetric expansion of the gap genes is an important feature of our dataset that can be used to challenge computational models of gap gene pattern formation and refinement (Jaeger et al., 2004a,b; Bieler et al., 2011; Hengenius et al., 2011; Papatsenko and Levine, 2011).

### Cell fates are canalized in *bcd* RNAi embryos

The *bcd* RNAi atlas provides a unique opportunity to examine how and when individual cells canalize cell fate following a strong genetic perturbation. Depletion of *bcd* leads to a complete replacement of the head and thorax with a second set of tail structures. This observation has been interpreted as strong canalization of cell fate because discrete structures still form (Nüsslein-Volhard et al., 1987). However, this canalization could either be mediated by the segmentation network or by later compensatory processes such as apoptosis. In support of the latter hypothesis, *bcd* RNAi and mutant cuticles are much smaller than the wild-type cuticle and there is extensive apoptosis in *bcd* mutant embryos, which has been interpreted as selective elimination of mis-specified cells (Werz et al., 2005).

To determine whether the segmentation network directly canalizes cell fate, we compared gene expression profiles of individual cells in *bcd* RNAi and wild-type embryos. The gene expression profile of a cell prefigures its eventual cell fate (Lehmann and Frohnhofer, 1989; Lawrence, 1992; St Johnston and Nüsslein-Volhard, 1992). All of the individual gene expression patterns in *bcd* RNAi are different from wild type, but patterns do not report directly on cell fate; rather, cell fate is a function of the transcriptome of each cell. Although we cannot yet measure the transcriptome of individual cells in intact embryos, we can use our dataset to analyze the co-expression of key segmentation genes in individual cells over 1 h of development.

**Fig. 5.**
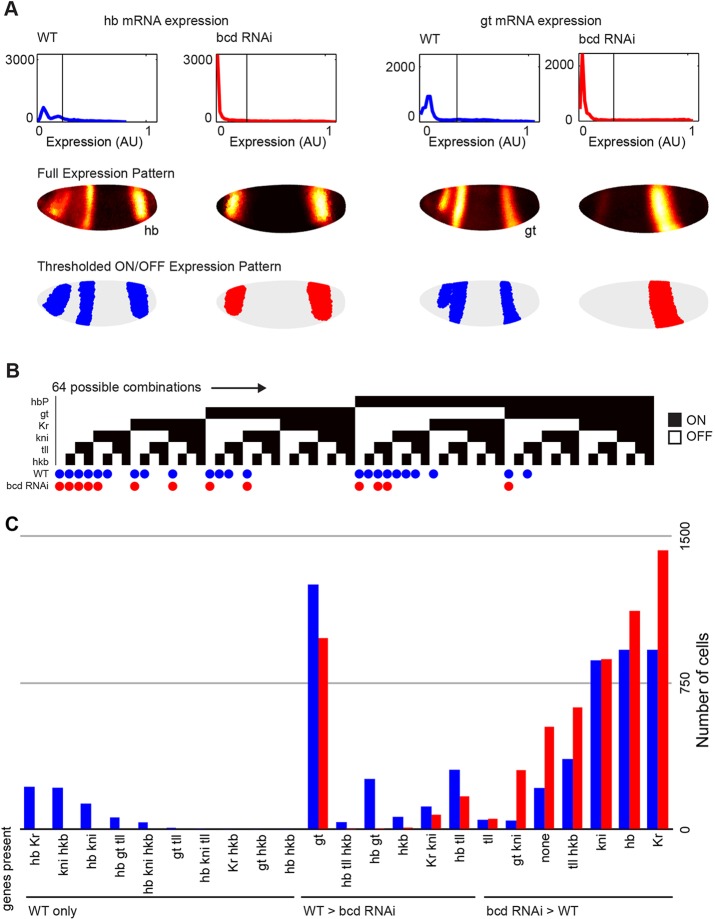
**There are no new combinations of gap and terminal gene expression patterns in *bcd* RNAi embryos.** (A) For each gene, we thresholded the expression pattern to find ON cells. Histograms of expression levels (top), heat maps of continuous expression patterns (middle) and the thresholded pattern (bottom). (B) Each column represents one of the 64 possible ON/OFF combinations of six genes. Filled squares indicate presence of a TF in a combination. There are 23 combinations present in wild type (indicated by blue dots) and 13 combinations present in *bcd* RNAi (red dots). No combinations are present only in *bcd* RNAi. (C) The number of cells with each combination in each genotype.

To determine whether new cell fates emerge in *bcd* RNAi embryos, we need to decide what constitutes a significant difference. It is not clear what quantitative changes in relative amounts of mRNA would result in a cell fate change, but certainly the emergence of a new combination of mRNAs in a single cell would indicate a new cell fate. We therefore defined cell fate as a binary gene expression profile where each gene is either ON or OFF; this is a course-grained definition that captures combinations of genes expressed in each cell. We analyzed combinations of genes in the first zygotic layer of the network: the gap and terminal genes *Kr*, *hb*, *gt*, *kni*, *tll* and *hkb*. For each of the six regulators, we thresholded expression to classify cells as ON or OFF (Fig. 5A, see Materials and Methods; supplementary material Table S4), giving 2^6^ (64) possible ON/OFF combinations.

By our simple definition, no new cell fates were created in *bcd* RNAi embryos. The third time point is illustrative of the general trend: of the possible 64 combinations, 23 cell fates were present in wild-type embryos (Fig. 5B). In wild type, there were no combinations with four or more genes and only four out of 20 possible combinations of three genes, consistent with the strong mutual repression between some of the gap genes (reviewed by Jaeger, 2011). All cells in *bcd* RNAi embryos belonged to 13 cell fates, all of which were present in wild type. For the 10 wild-type cell fates lost in *bcd* RNAi, most cells were located in the anterior (supplementary material Fig. S7). Virtually all of the shared cell fates changed in abundance between genotypes, with six more abundant in wild type, and seven more abundant in *bcd* RNAi (Fig. 5C; supplementary material Fig. S8). We also compared gene expression profiles using Hb protein in place of *hb* mRNA because these patterns differ (supplementary material Fig. S4). We again found that no new combinations arose in *bcd* RNAi (supplementary material Fig. S9). We conclude that the dramatic changes in gap gene expression patterns result from changes in the proportion of cells with wild-type fates. This result supports the hypothesis that the first zygotic layer of the segmentation network directly canalizes cells towards normal fates.

Canalization is also observed for other ON/OFF thresholds and time points. For T=3, over a wide range of ON/OFF thresholds, we found that all combinations of genes in *bcd* RNAi were also present in wild type (supplementary material Fig. S8). When we used Hb protein in our analysis, there were no new combinations within a more limited range of thresholds, because the Hb protein data is harder to faithfully partition into ON and OFF cells (supplementary material Fig. S9). At other times and thresholds, we sometimes found a handful of cells with a combination unique to *bcd* RNAi, but in virtually all cases, this combination existed in wild type at other thresholds or adjacent time points. These failures to detect combinations in wild type likely arose from the higher background signal for some genes in the wild-type expression data (Fig. 5A). At T=6 in *bcd* RNAi, this analysis detected a handful of cells with new combinations of the terminal patterns of *tll*, *hkb* and *Kr*, but this effect is likely to be an artifact of the low quality wild-type T=6 *hkb* data, as visual inspection revealed these patterns overlap in wild type (supplementary material Fig. S10C).

To guard against the possibility that the fine registration using the *ftz* stain influenced our interpretation of the data, we repeated this analysis on a coarsely aligned atlas where embryos are aligned without the *ftz* fiduciary marker, using only morphology (supplementary material Fig. S10). For T=1-3, there were no additional combinations over the full range of thresholds. For T=4-6 in *bcd* RNAi, the analysis detected three additional combinations of *hb*, *tll*, *hkb* and *Kr*, each with one or two cells at the boundaries of these patterns (supplementary material Fig. S10). We conclude that the fine registration did not confound our interpretation that cell fate is canalized in *bcd* RNAi embryos.

### The pair-rule gene expression patterns of *eve* and *ftz* are dynamically canalized in *bcd* RNAi embryos

**Fig. 6.**
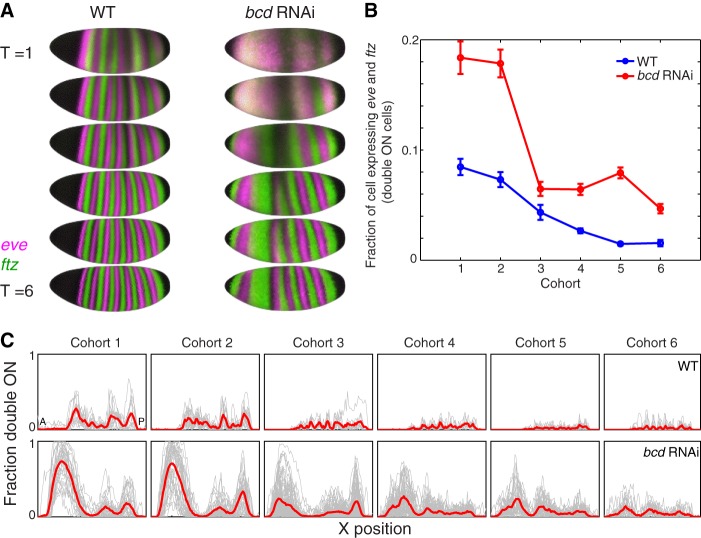
**The mRNA expression patterns of *eve* and *ftz* canalize over the blastoderm stage.** (A) *eve* (pink) and *ftz* (green) mRNA patterns in the gene expression atlas for each cohort in wild type and *bcd* RNAi. Cells with no expression appear black and cells expressing both *eve* and *ftz* appear white. (B) We quantified the fraction of cells that express both *eve* and *ftz* in individual embryos. For each embryo, we thresholded each expression pattern to be ON or OFF, and counted the fraction of cells where both genes were ON. Wild type, *n*=113; *bcd* RNAi, *n*=287. (C) Most cells expressing both *eve* and *ftz* are located in the anterior of the embryo. In each panel, the fraction of cells expressing both *eve* and *ftz* in each 1% egg length along the anterior posterior axis is plotted. Individual embryos are plotted in gray and population averages are plotted in red. The double ON cells in the posterior in the vicinity of stripe 7 in cohort 2 likely result from the changes in the gap gene expression patterns that affect *eve* and *ftz* differently.

The primary pair-rule genes *eve* and *ftz* define the parasegment boundaries that later establish the compartment boundaries (Martinez-Arias and Lawrence, 1985; Lawrence, 1992). We chose to examine this layer of the network separately from the gap and terminal genes for three reasons: (1) *eve* and *ftz* are regulated by both the gap and maternal genes; (2) these genes may be sensitive to quantitative changes in relative levels of the gap genes not detected by our binary combination analysis; and (3) while the initial gap gene patterns appear in stage 4, before we started collecting data, our stage 5 data captured the emergence and refinement of *eve* and *ftz* expression. In wild type, these two gene expression patterns are mutually exclusive for virtually the entire blastoderm stage (Fig. 6). In *bcd* RNAi, some individual embryos had extensive overlap of these two patterns. To quantify this difference, we examined individual embryos stained for *eve* and *ftz*, thresholded each gene separately to be ON or OFF, and counted the fraction of cells with both genes ON. In *bcd* RNAi embryos in the first two cohorts, ∼20% of cells expressed both *eve* and *ftz*, substantially more than the <10% seen in wild type*.* Beginning with the third cohort, the fraction of cells in *bcd* RNAi embryos expressing both genes dropped sharply (Fig. 6B, supplementary material Fig. S11). The shape of the trend does not depend on the threshold used to assign cells as ON and OFF, or the *in situ* hapten (supplementary material Fig. S11). In our dataset, early *eve/ftz* overlap resolves into mutually exclusive stripes, another manifestation of canalization in the segmentation network.

## DISCUSSION

We used the maternal Gal4 shRNA system to build a gene expression atlas of an embryo depleted of *bcd*, a maternally deposited transcription factor crucial for anterior-posterior patterning of the *Drosophila melanogaster* embryo. This is the first 3D cellular resolution atlas of a genetic perturbation. The technical innovations we describe can be readily applied to build gene expression atlases of additional genetic perturbations. The specific dataset we present will be broadly useful for testing computational models of the segmentation network. Our data also reveal that the segmentation network directly canalizes cell fates: there were no new combinations of gap and terminal gene expression following *bcd* depletion. In the next layer of the network, the pair-rule genes *eve* and *ftz* initially overlapped, but eventually established sharp parasegment boundaries. We conclude that the anterior-posterior patterning network robustly specifies cell fates following the loss of a key maternal input.

### Extension of the technique and utility of the dataset

Imaging techniques are uniquely positioned to capture how development unfolds in space and time (Megason, 2009). Gene expression atlases combine the spatiotemporal expression patterns of many genes in the same morphological framework, enabling network-level computational analyses (Jaeger et al., 2004b; Janssens et al., 2006; Papatsenko and Levine, 2008; Manu et al., 2009a,b; Bieler et al., 2011; Gursky et al., 2011; Kozlov et al., 2012; Ilsley et al., 2013; Kim et al., 2013). Although imaging of single embryos is currently limited to four or five genes (Kosman et al., 2004; Dubuis et al., 2013a), atlases can overcome this limit by registering together data from many individual embryos stained for different genes but with a common fiduciary marker. This method is flexible and extendable: adding genes to the dataset simply requires co-staining with the fiduciary marker and imaging at high-resolution on a two-photon microscope. The software for image processing, atlas building, data visualization and analysis are freely available (http://bdtnp.lbl.gov). Here, we extended atlas-building methods to genetically perturbed embryos, overcoming multiple technical challenges: collecting sufficient numbers of embryos, reducing population variability and building the appropriate registration template.

In the long term, other spatially and temporally resolved methods for quantitating gene expression may emerge (reviewed by Crosetto et al., 2015). For example, mRNA sequencing can be performed on cryo-sliced embryos (Combs and Eisen, 2013) or *in situ* (Lee et al., 2014), though the latter remains prohibitively expensive for most labs. In the meantime, gene expression atlases are an accessible technique for examining the ensemble behavior of gene regulatory networks in single cells. By combining data for many genes into a unified morphology, atlases enable computational modeling and analysis. In particular, we anticipate that the asymmetric expansion of expression patterns in the *bcd* RNAi embryo will provide a useful challenge for computational models of the gap gene network.

### Phenotypic variability in *bcd* RNAi embryos can be controlled, and may be useful in the future

To build the gene expression atlas, we reduced the variability in the distribution of phenotypes in *bcd* RNAi embryos using specific collection conditions and manual curation. In the future, variability may be useful for studying other emergent properties of the network. For example, partially penetrant mutants helped constrain mathematical models of signal integration (Corson and Siggia, 2012). Some of our phenotypic variability may stem from inconsistent shRNA knockdown (Mohr and Perrimon, 2012), but the mutant data suggest much of the variability must emerge from the network response to *bcd* depletion (Fig. 1C)*.* Increased variability in mutant phenotypes is common (Waddington, 1942; Wieschaus et al., 1984), and recent examination of gene expression patterns in *tll*, *Kr*, *kni* and *Kr/kni* mutants concluded that there was more molecular variability in mutant embryos than in wild-type embryos (Janssens et al., 2013; Surkova et al., 2013). In *bcd* RNAi embryos, the variability in anterior *eve* stripe expression may explain the distribution of cuticle denticle bands. To enable the study of this variability, we have made the data from individual embryos with split anterior *eve* stripes publicly available on FigShare at http://dx.doi.org/10.6084/m9.figshare.1270915.

### The segmentation network canalizes cell fate in *bcd* RNAi embryos

Although it was known that cell fates were canalized in *bcd* mutant embryos at the time of hatching, it was not clear whether this canalization occurred immediately, due to the segmentation network, or later, due to downstream compensatory processes. The extensive apoptosis in *bcd* mutant embryos was proposed to be due to removal of mis-specified cells (Werz et al., 2005). Misspecification can either imply the presence of too many cells of a given type, or the emergence of new types. Our analysis provides direct evidence that the segmentation network prevents the creation of new cell fates in the absence of a maternal input. Several lines of evidence predicted this canalization, including cytoplasmic transplantation experiments (Nüsslein-Volhard et al., 1987), the coordinated shifts in gene expression patterns following changes in *bcd* dose (Driever and Nüsslein-Volhard, 1988; Struhl et al., 1989; Liu et al., 2013) and the molecular canalization of gene expression patterns in wild type (Manu et al., 2009a,b; Gursky et al., 2011). We have shown that canalization occurs early and strongly, resulting in changes in the abundance of most cell fates, but not the creation of new fates. This canalization is likely enforced by the abundant cross-repression in the gap gene network (Jäckle et al., 1986; Kraut and Levine, 1991b; Jaeger et al., 2004b; Jaeger, 2011; Papatsenko and Levine, 2011; Sokolowski et al., 2012).

We propose that the increased apoptosis in *bcd* mutant embryos does not eliminate cells with new fates, but instead compensates for enlarged compartments. The *eve* and *ftz* stripes set compartment size, and small compartments experience reduced cell death, whereas large compartments experience increased cell death (Namba et al., 1997; Hughes and Krause, 2001). The wide second *ftz* stripe (Figs 2 and 6) is approximately where the most apoptosis is observed in *bcd* mutant embryos (Werz et al., 2005). According to our analysis, cells undergoing apoptosis do not have new fates at the blastoderm stage. Rather, they reside in a compartment that is too large, and this increased compartment size may trigger cell death. Planarians also induce cell death in enlarged tissues (Pellettieri et al., 2010). Numerous mechanisms have been proposed for how tissues measure their size, including secreted signals, surface to volume ratio or mechanical tension (Umulis and Othmer, 2013). In *Drosophila* embryos, cells on the compartment boundaries express the secreted signaling molecules Wingless and Hedgehog (Lawrence, 1992), which could establish compartment size; this mechanism would predict that apoptosis is enriched in the center of the compartment.

### Dynamic canalization establishes sharp *eve* and *ftz* parasegment boundaries

Even though our analysis of combinations of gap gene expression indicated that there were no new cell fates, we observed significant differences in expression of two gap gene targets: *eve* and *ftz* (Fig. 6). At the first two time points, ∼20% of cells in *bcd* RNAi embryos express both *eve* and *ftz*, but this fraction later plummets as the patterns resolve into mutually exclusive stripes. The majority of co-expressing cells are in the anterior, as expected, but a small fraction overlap *eve* stripe 7, the most variable of the *eve* stripes (Dubuis et al., 2013b). Similar early overlaps of *eve* and *ftz* that resolve to mutually exclusive stripes have recently been reported in *Kr* mutant embryos (Surkova et al., 2013).

We speculate on two possible causes of increased *eve/ftz* co-expression in *bcd* RNAi embryos. First, changes in relative amounts of gap gene expression may impact early *eve* and *ftz* expression differently. Each gap gene can regulate targets in a concentration-dependent way (Clyde et al., 2003; Yu and Small, 2008; Dubuis et al., 2013b). Therefore, a cell may express the same combination of genes, but their relative amounts may differ, leading to changes in target gene expression. Second, subtle changes in the dynamics of gap gene expression may affect *eve* and *ftz* differently, leading to early co-expression. The limited kinetic data in our atlas may not be sufficient for capturing such differences. Emerging experimental methods that can measure absolute amounts of mRNA and the dynamics of mRNA production in individual cells (Garcia et al., 2013; Little et al., 2013) will be useful for deciphering the cause of *eve/ftz* co-expression.

The resolution of *eve* and *ftz* boundaries is likely mediated by direct repression of *ftz* by *eve* and indirect repression of *eve* by *ftz* through seven-stripe enhancers (Jiang et al., 1991; Manoukian and Krause, 1992; Fujioka et al., 1996; Saulier-Le Drean et al., 1998; Nasiadka and Krause, 1999; Schroeder et al., 2011). This canalization of compartment boundaries may be a general feature of the network response to mutants.

### Conclusions

Re-examining a classic perturbation at cellular resolution provided direct evidence that the segmentation network canalized cell fates early and robustly. Our increased resolution also revealed quantitative features of the network response to perturbation, including the asymmetric expansion of the gap genes and the dynamic canalization of the parasegment boundaries. The *bcd* RNAi gene expression atlas will provide the developmental systems biology community with a cellular resolution dataset for testing computational models of how individual regulatory circuits position expression domains (Staller et al., 2015). These studies also lay important groundwork for our long-term goal of identifying the features of the network architecture that contribute to canalization of cell fate.

## MATERIALS AND METHODS

### Fly work

We depleted *bcd* with *UAS-shRNA-bcd* (TRiP GL00407) and the *maternal triple driver Gal4 *(*MTD-Gal4*)** (Fig. 1A). For reference, we used *bcd^12^* (Bloomington 1755) (Frohnhöfer and Nüsslein-Volhard, 1986; Struhl et al., 1989). For controls we used *maternal-tubulin-Gal4* (*mat-tub-Gal4*), GL01320 *UAS-shRNA-bcd* and TB184 *UAS-shRNA-GFP* (supplementary material Fig. S2) (Neumuller et al., 2012; Staller et al., 2013). For future work with other maternal effect genes, we recommend *mat-tub-Gal4* (supplementary material Fig. S2). We crossed virgin *MTD-Gal4/UAS-shRNA-bcd* females to males homozygous for reporter constructs. Enhancers were cloned into the *Not*I and *Bgl*II sites of *pBOY-lacZ* and integrated in attP2 (Groth et al., 2004). Reporter sequences, original references and cloning primers are listed in supplementary material Table S1.

### Preparation of unhatched larval cuticles

Unhatched larval cuticles were mounted in lactic acid (Stern and Sucena, 2000). We manually counted the number of denticle bands on each cuticle under dark field, rounding up partial segments. For the majority of cuticles shown, a *z*-stack of two to four images was computationally flattened with Helicon Focus (Helicon Soft).

### Quantitative RT PCR

Embryos were collected for 2 h and snap frozen in liquid nitrogen. We extracted RNA with Trizol and synthesized cDNA with superscript reverse transcriptase (Life Technologies). We used TaqMan probes (Life Technologies) with *actin* as a reference.

### *In situ* hybridization

All RNA stains were performed as described previously (Fowlkes et al., 2011; Wunderlich et al., 2014). Briefly, embryos were collected over 4 h at 25°C, dechorionated in bleach, fixed in formaldehyde/heptane for 25 min, dehydrated with methanol and stored in ethanol at −20°C. We used a digoxigenin (DIG) *ftz* probe, a dinitrophenol (DNP) probe against the gene of interest, and developed them sequentially with a tyramide amplification reaction (PerkinElmer), with DIG in the coumarin channel and DNP in the Cy3 channel. We kept the amplification in the linear range, as described previously (Wunderlich et al., 2012). After RNase treatment overnight at 37°C, DNA was stained with Sytox Green (Life Technologies). Embryos were dehydrated with ethanol, cleared with xylenes and mounted in DePeX (Electron Microscopy Sciences). To acquire Hb protein data, we stained embryos first with *ftz* DNP in the coumarin channel, then with guinea pig anti-Hb [a generous gift from John Reinitz (Chicago, IL, USA)] and with goat anti-guinea pig AlexaFluor 555 (Life Technologies).

### Image acquisition and manual data curation

We acquired *z*-stacks with two-photon excitation at 750 nm, with 1 μm increments and simultaneously collected the three fluorescent channels. Protein stains were imaged in the same way. We use automated image processing to segment the nuclei and extract expression of the two genes in every cell, creating a pointcloud file for each embryo (Luengo Hendriks et al., 2006). We manually classified embryos into six cohorts: 0-3%, 4-8%, 9-25%, 26-50%, 51-75% and 76-100% membrane invagination, which evenly divide the ∼60 min blastoderm stage (Keränen et al., 2006). To remove individual embryos with weak phenotypes from the set of embryos laid by old mothers, we manually inspected the *ftz* pattern. For time points 4-6, we removed embryos with a narrow second *ftz* stripe or an extra *ftz* stripe. For *eve* stains, we removed embryos with a split anterior stripe.

### Finding expression pattern boundaries

Pointcloud files were manipulated in MatLab (MathWorks) using the pointcloud toolbox (bdtnp.lbl.gov). For each embryo, we created line traces for 16 strips around the dorsal ventral axis, and found the inflection point in each trace (the egglengthnormalize, rotation, align, stretch, extractpattern, segmentgap, getstrips, and locateapboundaries tools). Similar results were obtained when we used the half maximum of each line trace (with the locateapboundaries tool).

### Building the *bcd* RNAi gene expression atlas

To account for a small increase in cell number and changes in cell density, we built a new morphological template for the *bcd* RNAi atlas using 1567 embryos (Fowlkes et al., 2008, 2011). To build a new gene expression registration template, we used 249 embryos stained with only DNP *ftz* probes. Embryo alignment is a two-step process: first embryos are aligned coarsely to the morphological template; second they are finely aligned to the registration template using the DIG *ftz* gene expression pattern. This fine scale alignment involves a local warping of each embryo described in detail previously (Fowlkes et al., 2008). The degree of local warping tends to be higher at later time points when the patterns are sharper, leading to more reduction in variance (see Fowlkes et al., 2008). Each gene was normalized separately so that relative levels between time points were preserved, but the absolute levels between atlases are likely different. Cell density maps (supplementary material Fig. S3) were generated using the demo_densities function in the pointcloud toolbox.

We have provided the *bcd* RNAi gene expression atlas and a bundled file containing all the individual embryos stained for *eve* and *ftz*, including those that were excluded from the atlas on FigShare (http://dx.doi.org/10.6084/m9.figshare.1270915).

### Identifying combinations of ON and OFF cells

We thresholded *Kr*, *hb*, *kni*, *gt*, *tll* and *hkb* mRNA at each time point by creating a histogram, finding the peak of the OFF cell population and adding one standard deviation (e.g. supplementary material Fig. S11B, Table S4). For *eve* and *ftz* co-expression, we determined thresholds for each gene in each embryo and recorded the fraction of cells expressing both. Using published wild-type embryos, we found that swapping the haptens (DNP/DIG) did not change the fraction of double ON cells (supplementary material Fig. S11A).

## Supplementary Material

Supplementary Material
