# Supplementary material for: A gene expression atlas of a bicoid-depleted Drosophila embryo reveals early canalization of cell fate
Source: Development. 2015 Feb 1;142(3):587–96. doi: 10.1242/dev.117796 (PMC4302997; doi:10.1242/dev.117796)
Supplement: Supplementary Material [file supp_142_3_587__index.html]

Supplementary Material 

# A gene expression atlas of a *bicoid*-depleted *Drosophila* embryo reveals early canalization of cell fate

## DEV117796 Supplementary Material

**Files in this Data Supplement:**

- Supplementary Material
