# Supplementary material for: A gene expression atlas of a bicoid-depleted Drosophila embryo reveals early canalization of cell fate
Source: Development. 2015 Feb 1;142(3):587–96. doi: 10.1242/dev.117796 (PMC4302997; doi:10.1242/dev.117796)
Supplement: Supplementary Material [file supp_142.3.587_DEV117796supp.pdf]

Supplementary Figure Legends

Fig. S1: The phenotype spectrum of *bcd* RNAi cuticles resembles an allelic series

A. Unhatched cuticles of embryos laid by *MTD-Gal4/UAS-shRNA-bcd* females display variable numbers of denticle bands. A representative individual from each class is shown. The filzkörper, a tail structure, is indicated with arrow heads. Cuticles with weak *bcd* phenotypes have 9 or more bands and some thoracic segments, but no head structures. Cuticles with strong *bcd* phenotypes have only abdominal segments and a duplicated filzkörper. Scale bar 200 microns.
B. An additional *UAS-shRNA-bcd* (GL01320) line gives a similar phenotype.
C. The maternal driver *mat-tub-Gal4* gives a similar phenotype with both *UAS-shRNA-bcd* lines.

To test viability, we arrayed 200 embryos on an agar plate and counted the number hatched after 48 hrs at 25C. For *bcd* RNAi embryos, 0/200 and 0/200 embryos hatched; for embryos laid by *MTD-Gal4/UAS-shRNA-GFP* females 222/239 and 178/200 of embryos hatched (TRiP Toolbox stock 182) (Neumuller et al., 2012).

Fig. S2: Mother age contributes strongly to the strength and variability of the *bcd* RNAi phenotype while temperature, *UAS-shRNA-bcd* line, maternal driver, zygotic *UAS-shRNA-bcd* construct copy number, and paternal genotype do not.

A. The severity of *bcd* phenotype increases as the flies age. We allowed *MTD-Gal4/UAS-shRNA-bcd* flies to eclose for 48 hrs and counted denticle bands every few days. Old mothers were ≥11 days old and embryos were collected for the gene expression atlas. Counts: Day 2 n = 137, Day 6 n = 173, Day 8 n = 224, Day 11 n = 86, Day 14 n = 127, Day 16 n = 58.
B. qPCR indicates >75% knockdown in embryos laid by older mothers. Embryos were collected for 2 hrs. As a reference for WT *bcd* levels we used embryos from Day 7 *MTD-Gal4/UAS-shRNA-GFP* embryos. Samples have three replicates (two replicates for Day 10) and error bars are SEM.
C. Paternal genotype does not meaningfully influence the *bcd* RNAi phenotype. *MTD-Gal4/UAS-shRNA-bcd* virgin females were crossed to males homozygous for *UAS-shRNA-bcd* or with males homozygous for a enhancer lacZ reporter (WT). Progeny from the first cross will have 1-2 copies of the *UAS-shRNA-bcd* construct (blue), while progeny from the second cross will have 0-1 copies of the *UAS-shRNA-bcd* construct (red). We could not detect a difference between the number of ventral denticle bands visible in each populations (p values from Kolmogorov-Smirnov test). Note that the effect of mother age is much greater than paternal genotype (compare Day 1 and Day 6 samples). Although it is possible that shRNAs against *bcd* are zygotically expressed, they do not meaningfully contribute to the phenotype, consistent with the purely maternal effect of *bcd*. These cross also showed there was no detectable paternal effect.
D. Two *UAS-shRNA-bcd* lines yield similar phenotypes. We tested two shRNA lines (GL00407 and GL01320) at 25°C and 29°C, taking samples on Day 4 and Day 10. For these crosses we used the *mat-tub-Gal4* maternal driver; this driver tends to give a more consistent phenotype than *MTD-Gal4*. Analysis of variance (ANOVA) of temperature, UAS-line, and day reveal that each has a significant but small effect, less than one segment in all cases. For the *mat-tub-Gal4* driver, the distribution of phenotypes laid by young mothers approaches the steady state distribution seen for old mothers with *MTD-*

Gal4 more quickly. This result is consistent with more uniform phenotypes for the *mat-tub-Gal4* driver with shRNAs against other genes (Staller et al., 2013). The distributions of embryos laid by old mothers of both genotypes are comparable. For future work depleting other maternal effect genes, we recommend the *mat-tub-Gal4* driver.

Fig. S3: *bcd* RNAi embryos have more cells and altered cell density patterns.

A. Average cell density maps of WT and *bcd* RNAi embryos. While the physical shape of the embryos remains asymmetric, the posterior density pattern is duplicated in the anterior of *bcd* RNAi embryos, like some mRNA patterns. Embryos from stage 5:51-100% (time points 5 and 6 in the gene expression atlases) are shown. Note these images sometimes do not load in Preview and are best viewed in Adobe Acrobat.
B. Histogram of cell counts in *bcd* RNAi embryos and WT (transgenic) embryos.

Fig. S4: The *bcd* RNAi gene expression atlas perturbs *hb* mRNA and protein levels.

A. Hb protein expression pattern changes over stage 5 in both WT and *bcd* RNAi. In WT both maternal *hb* mRNA and *bcd* activated zygotic mRNA contribute to the anterior pattern, while in *bcd* RNAi, only maternal mRNA contributes to the early, broad anterior pattern (Tautz, 1988). Note each atlas is normalized separately, so absolute levels are not comparable between atlases. Relative levels change extensively.
B. In both WT and *bcd* RNAi, *hb* mRNA (gray) and protein (red) patterns are different.

Fig. S5: The boundaries of the *eve* stripes move over stage 5 in *bcd* RNAi embryos.

A. Boundary positions calculated using inflection points of individual embryos. Stripe 4 and 5 appear in a handful of embryos is cohort 4, but not frequently enough to reliably quantify boundary position. In each plot, anterior is left, dorsal is top.
B. The widths of the *eve* stripes contract in *bcd* RNAi embryos. At T = 5 *eve* stripes 4-7 are approximately 1.7, .6, 1.4, and 1.3 cell widths wider in *bcd* RNAi than WT. At T = 6 eve stripes 4-7 are approximately 1.3, .6, 1.5, and .3 cell widths wider in *bcd* RNAi than WT. Data calculated from one DV strip along the left side of the embryo. Error bars are SEM.

Fig. S6: The coefficient of variation of most of the gap and *eve* stripe mRNA pattern widths are similar between WT (blue) and *bcd* RNAi (red). The exceptions are *Kr* and the anterior *eve* stripe, which are more variable in *bcd* RNAi embryos. The ventral region of the ectopic anterior hb pattern (DV strips 7-11) is very faint in *bcd* RNAi embryos, and our analysis script struggles to reliably find a boundary, so this analysis likely overstates the variability in this region. Pattern widths calculated with the inflection point, but using the half maximum led to very similar measurements. The ectopic anterior *hb* pattern in *bcd* RNAi is compared to the *hb* posterior pattern in WT.

Fig. S7: The locations of each transcription factor combination at T=3 in WT (blue) and bcd RNAi (red). The “sum” of each catagory is shown at the bottom.

Fig. S8: Changing the ON/OFF threshold does not meaningfully change the conclusions of the combination analysis.

A. For a range of thresholds and most time points, all combinations present in *bcd* RNAi also present in WT. See Fig. S9B for schematic of how we vary thresholds.

B. When a combination was detected as unique to *bcd* RNAi, this was generally because it was not detected in WT for that threshold and time point. At high thresholds, overlap between adjacent patterns (*Kr* and *kni* or *kni* and *gt*) were not detected in WT. At T=4, the adjacent *hb, hkb* and *tll* patterns do not overlap enough to be detected in WT at most thresholds, but this combination was found at T=3, so it is not a true new combination. The new combinations found at T=6 arise either because the WT *hkb* data is low quality or because the anterior duplicated *tll* domain is smaller than the posterior domain.
C. Line traces of *Kr*, *hkb*, and *tll* at T=6 in WT and *bcd* RNAi. Anterior-posterior position is on the x-axis and expression level is on the y-axis. The high levels of background in the WT *hkb* pattern may confound the combination analysis. The duplicated *tll* pattern in the anterior has weaker expression than in the posterior at T=6, which may explain the apparent emergence of the *Kr, hkb* combination. See also Fig. S10C.

Fig S9: Substituting Hb protein for *hb* mRNA does not meaningfully change the conclusions of the combination analysis

A. For a range of thresholds and most time points, all combinations present in *bcd* RNAi are also present in WT.
B. Compare to Fig. S8B. In practice, the Hb protein (hbP) data is more difficult to partition into ON and OFF cells. Our method for finding ON cells is to make a histogram of the expression data, find the peak of the OFF cells, and add one s.d. For Hb protein, we add 0.5 s.d. instead. Accordingly, in this table the threshold of 0.9 (or 1.1) means we used 0.9 (or 1.1) s.d. for the 5 mRNAs and 0.45 (or 0.55) for Hb protein. When using the Hb protein data, the analysis is more sensitive to changes in threshold. For example, at T=3 for a threshold of 1.2 (0.6 for Hb protein), the posterior Hb protein domain in WT is no longer detected, leading to the false detection of the *hbP, tll, hkb* combination in *bcd* RNAi.

Fig. S10: Repeating the combination analysis with a coarsely aligned atlas suggests the fine scale alignment using the fiduciary marker does not confound our conclusions.

A. For a range of thresholds and most time points, all combinations present in *bcd* RNAi also present in WT.
B. Compare to Fig. S9B. No new combinations arose for T=1-3. As expected, the abundances of many combinations changed subtly. New combinations are in bold. Most occurred with the fine atlas at other times or thresholds. e.g. *Kr, tll, hkb* in T=5 can be found in WT at T=6.
C. The mRNA patterns of *hb, Kr, tll,* and *hkb* at T=6 in WT and *bcd* RNAi. The combinations that were detected as unique to *bcd* RNAi in this time point are at the boundaries of these pattern in the termini. They may reflect subtle changes in the dynamics of terminal expression patterns, but are more likely artifacts of the differences in absolute levels between genotypes (which are not captured because each atlas is normalized separately) or because the T=6 *hkb* data has high background in WT, which causes this analysis to call it as ON in fewer cells.

Fig. S11: Thresholds, stain hapten, and mother age do meaningfully influence the fraction of cells expressing both *eve* and *ftz*.

A. The hapten (DIG or DNP) of the *eve* mRNA probe does not bias this analysis. *ftz* is always in the other channel.
B. A schematic shows the effects of varying the ON/OFF threshold for either *eve* or *ftz*. To find the threshold, we create a histogram of the expression level of each gene separately and identify the peak of the OFF cell population (mode). For our normal threshold, we add one standard deviation. We varied the threshold to be 0.6, 0.8, 1 and 1.2 standard deviations.
C. C-F) Regardless of the ON/OFF threshold used, the fraction of cells expressing both *eve* and *ftz* (double ON cells) decreases over time. The mixed mom *eve* DNP embryos (magenta) were collected from cages that had a wide range of mother ages before we started collecting from aged cages. We included these data because they have a very high quality stain. These data indicate that mother age does not have a meaningful effect on this analysis. In Fig. 6 the red and magenta data are combined. In all plots, error bars are the s.e.m.

Supplementary Figures

Figure S1

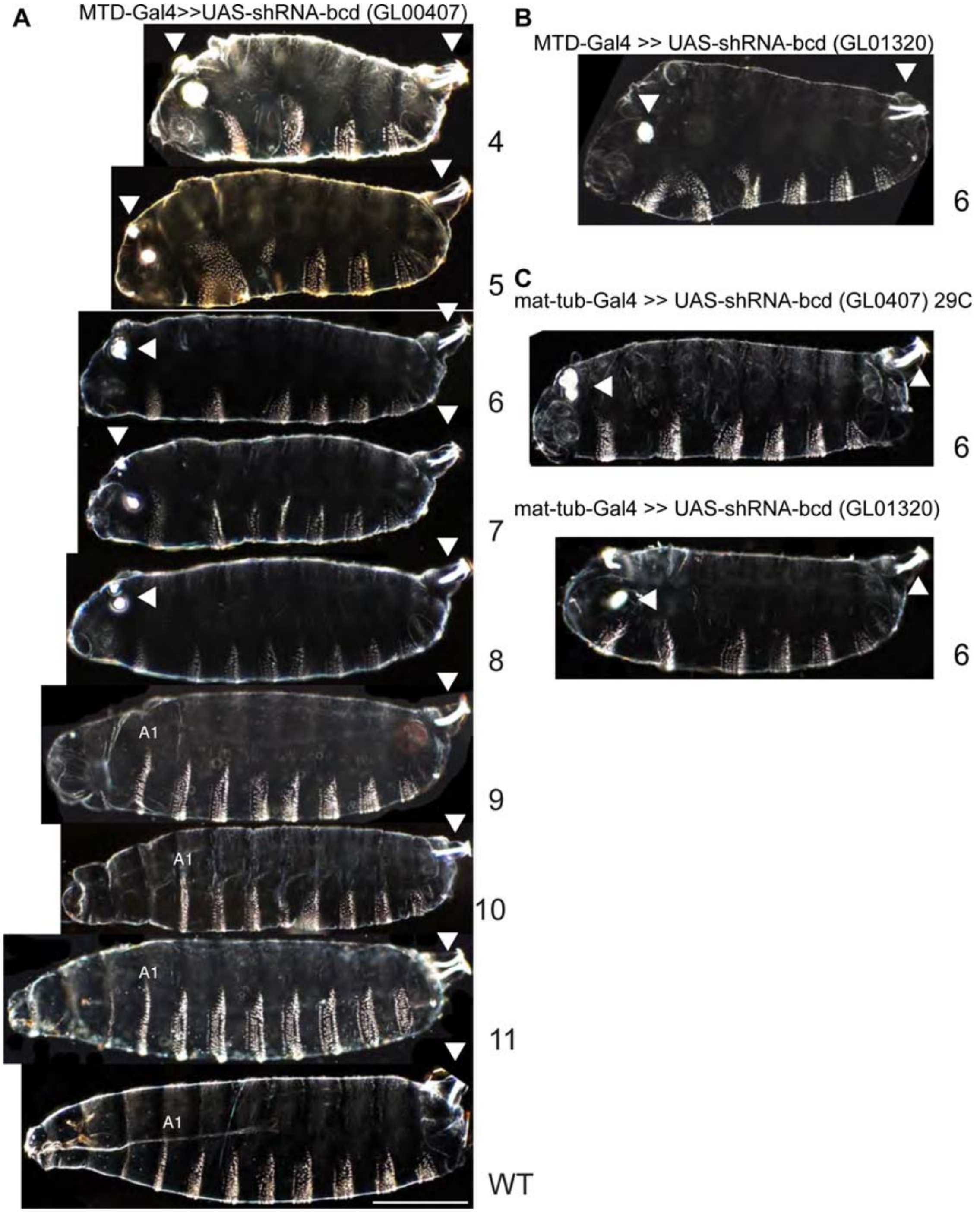

Figure S2

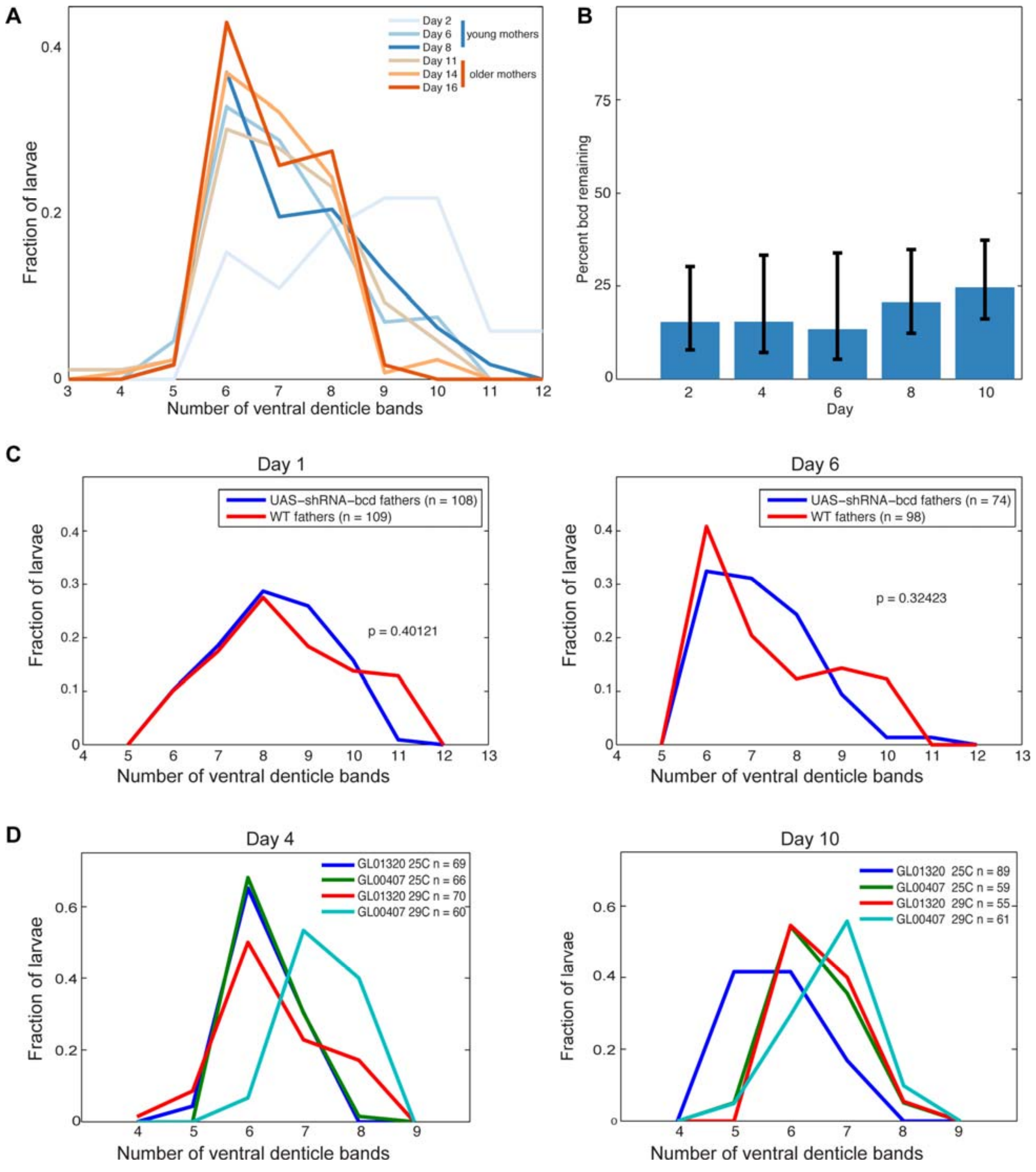
A
B
C
D
Day 2
Day 6
Day 8
young mothers
Day 11
Day 14
Day 16
older mothers
Fraction of larvae
Number of ventral denticle bands
Percent bcd remaining
Day
Day 1
Day 6
UAS–shRNA–bcd fathers (n = 108)
WT fathers (n = 109)
p = 0.40121
UAS–shRNA–bcd fathers (n = 74)
WT fathers (n = 98)
p = 0.32423
Day 4
Day 10
GL01320 25C n = 69
GL00407 25C n = 66
GL01320 29C n = 70
GL00407 29C n = 60
GL01320 25C n = 89
GL00407 25C n = 59
GL01320 29C n = 55
GL00407 29C n = 61

Figure S3

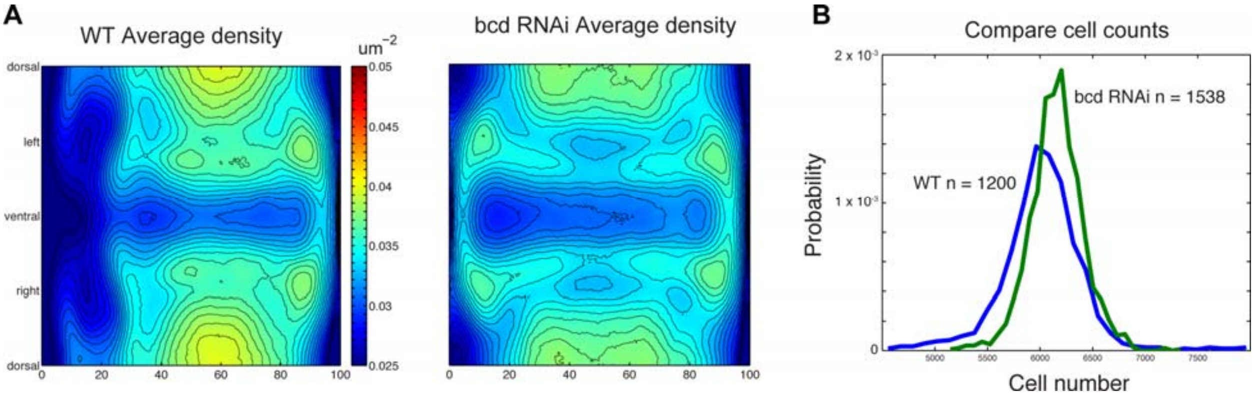

Figure S4

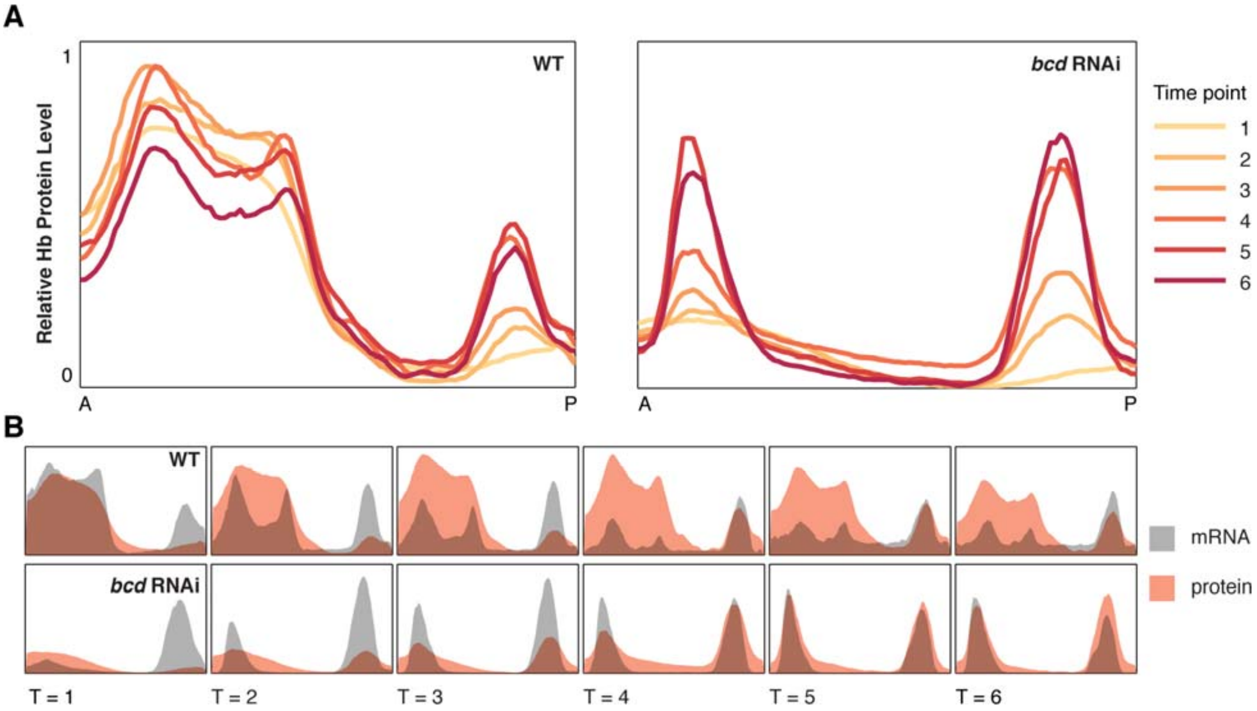

Figure S5

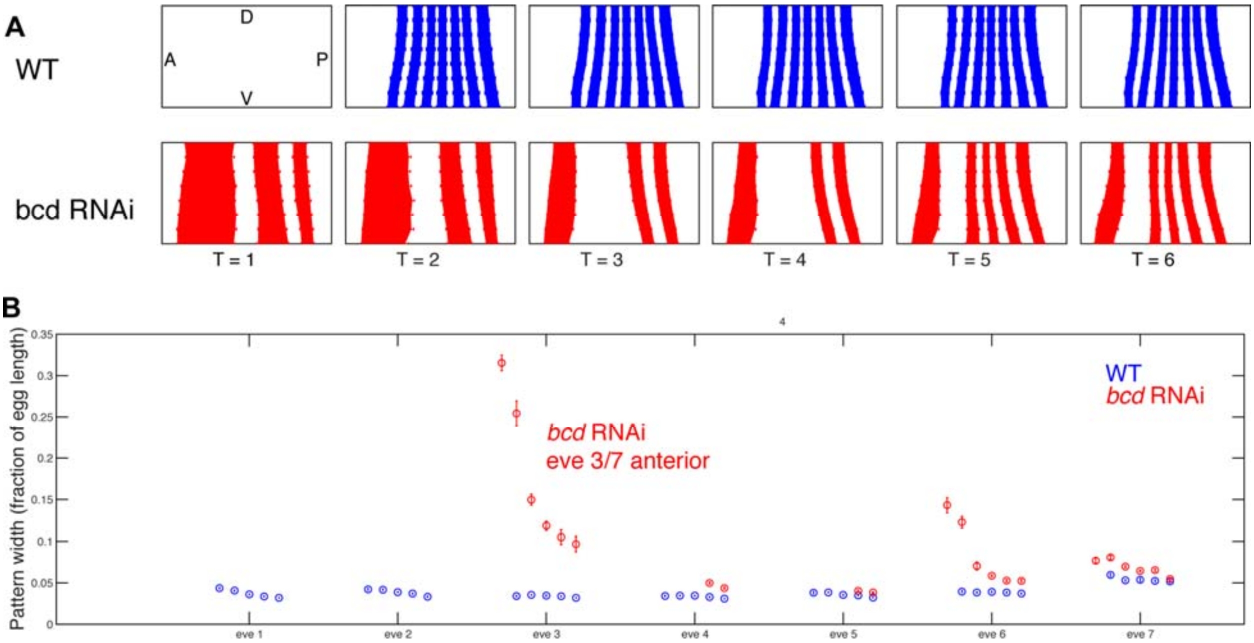
A
WT
D
A
P
V
bcd RNAi
T = 1
T = 2
T = 3
T = 4
T = 5
T = 6
B
Pattern width (fraction of egg length)
WT
bcd RNAi
bcd RNAi
eve 3/7 anterior
eve 1
eve 2
eve 3
eve 4
eve 5
eve 6
eve 7

Figure S6

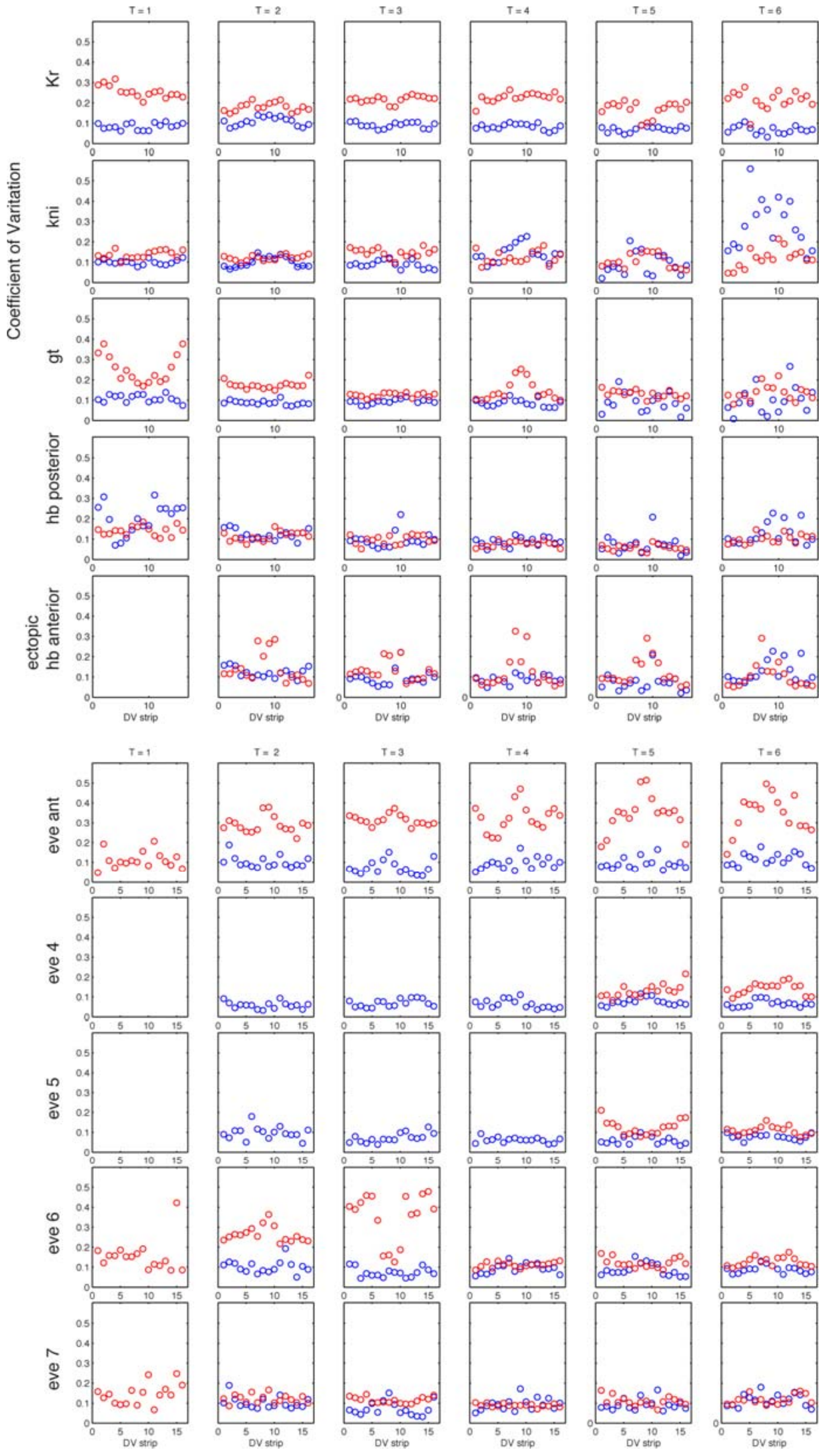
T = 1
T = 2
T = 3
T = 4
T = 5
T = 6
Coefficient of Variation
Kr
kni
gt
hb posterior
ectopic
hb anterior
DV strip
eve ant
eve 4
eve 5
eve 6
eve 7

Figure S7

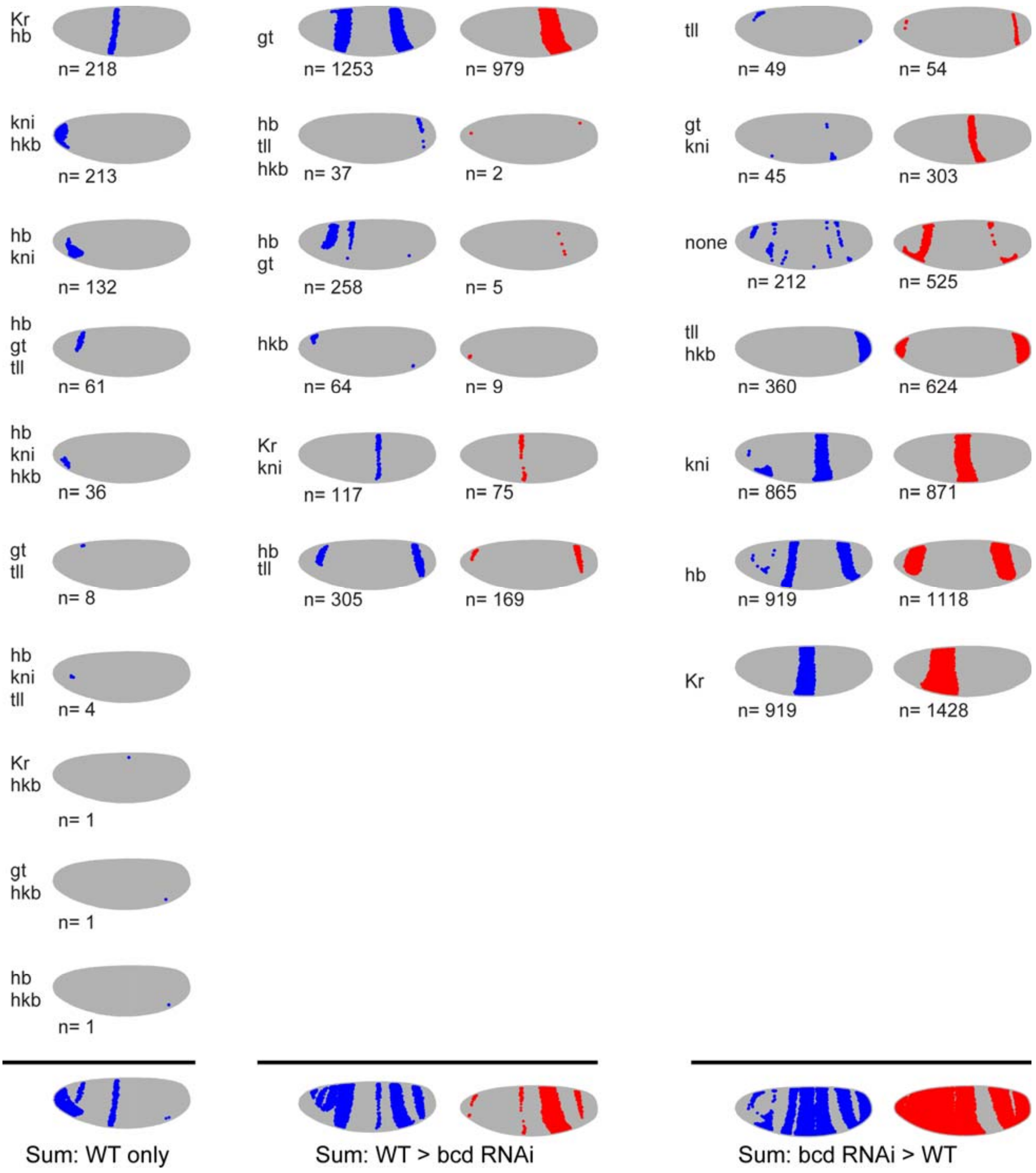
Kr hb
n= 218
kni hkb
n= 213
hb kni
n= 132
hb gt tll
n= 61
hb kni hkb
n= 36
gt tll
n= 8
hb kni tll
n= 4
Kr hkb
n= 1
gt hkb
n= 1
hb hkb
n= 1
gt
n= 1253
n= 979
hb tll hkb
n= 37
n= 2
hb gt
n= 258
n= 5
hkb
n= 64
n= 9
Kr kni
n= 117
n= 75
hb tll
n= 305
n= 169
tll
n= 49
n= 54
gt kni
n= 45
n= 303
none
n= 212
n= 525
tll hkb
n= 360
n= 624
kni
n= 865
n= 871
hb
n= 919
n= 1118
Kr
n= 919
n= 1428
Sum: WT only
Sum: WT > bcd RNAi
Sum: bcd RNAi > WT

Figure S8

A

Number of Gap Gene Combinations Unique to bcd RNAi (fine atlas)

Threshold	T = 1	T = 2	T = 3	T = 4	T = 5	T = 6
0.5	2	0	1	0	0	1
0.6	0	0	0	0	0	1
0.7	1	0	0	0	0	1
0.8	1	0	0	0	0	2
0.9	1	0	0	1	0	3
1	0	0	0	1	0	3
1.1	0	0	0	1	0	1
1.2	0	1	0	0	0	1
1.3	0	0	0	0	0	2
1.4	1	0	0	1	1	2
1.5	1	2	0	2	1	2

C

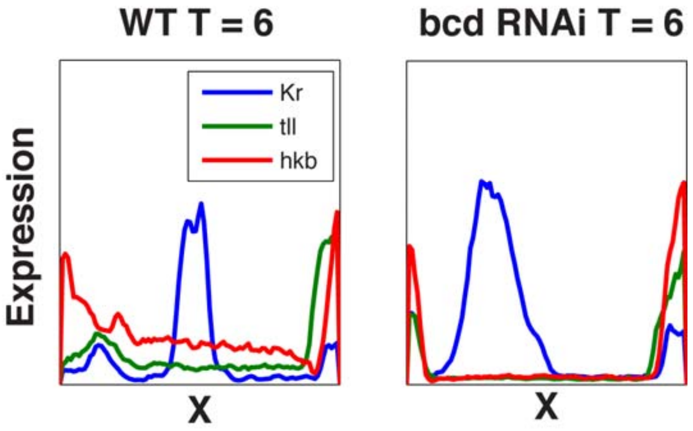

B **Identies and Counts of Gap Gene Combinations Unique to bcd RNAi—fine atlas**

Threshold	T = 1 Genes	T = 1 Count	T = 2 Genes	T = 2 Count	T = 3 Genes	T = 3 Count	T = 4 Genes	T = 4 Count	T = 5 Genes	T = 5 Count	T = 6 Genes	T = 6 Count
0.5	hb, gt, tll, hkb	13			none	90					hb, tll, hkb	5
	tll	10										
0.6											hb, tll, hkb	5
0.7	hkb	1									hb, tll, hkb	1
0.8	hkb	10									hb, Kr, tll, hkb	4
											hb, tll, hkb	2
0.9	hkb	9					hb, tll, hkb	10	hb, hkb	1	hb, tll, hkb	2
											hb, Kr, tll	1
											hb, Kr, tll, hkb	1
1							hb, tll, hkb	7			Kr, hkb	22
											hb, Kr, tll	1
											hb, Kr, tll, hkb	1
1.1							hb, tll, hkb	2			Kr, hkb	22
1.2			hb, hkb	1							Kr, hkb	34
1.3											Kr, kni	116
											Kr, hkb	39
1.4	hb, tll, hkb	216					gt, kni	98	Kr, kni	18	Kr, kni	90
											Kr, hkb	42
1.5	hb, tll, hkb	181	Kr, kni	13			gt, kni	60	Kr, kni	5	Kr, kni	65
			hb, tll, hkb	1			Kr, kni	16			Kr, hkb	41

Figure S9

Number of Gap Gene Combination Unique to bcd RNAi—hb protein

Threshold	T = 1	T = 2	T = 3	T = 4	T = 5	T = 6
0.5	2	0	0	2	1	3
0.6	2	0	0	1	0	4
0.7	2	0	0	1	0	4
0.8	2	0	0	0	1	4
0.9	2	0	0	0	2	3
1	1	0	0	0	1	1
1.1	0	0	0	0	1	1
1.2	1	1	1	1	2	3
1.3	2	1	1	0	2	5
1.4	1	1	1	3	3	4
1.5	1	2	1	4	3	4

Identies and Counts of Gap Gene Combinations Unique to bcd RNAi—hb protein

	T = 1		T = 2		T = 3		T = 4		T = 5		T = 6	
Threshold	Combo	Count	Combo	Count	Combo	Count	Combo	Count	Combo	Count	Combo	Count
0.5	gt, tll	51					Kr	878	Kr	922	Kr	932
	hbP, gt, tll, hkb	13					gt, tll	1			tll, hkb	45
											tll	1
0.6	gt, tll	88					gt, tll	1			Kr	1070
	hbP, Kr, kni	218									tll, hkb	87
											tll	3
											Kr, tll	1
0.7	gt, tll	69					gt, tll	1			Kr	1196
	hbP, Kr, kni	122									tll	8
											Kr, hkb	2
											Kr, tll	1
0.8	gt, tll	27							tll	2	Kr	1310
	hbP, Kr, kni	59									tll	14
											Kr, hkb	10
											Kr, tll	6
0.9	gt, tll	5							tll	10	tll	30
	hbP, Kr, kni	19							hkb	1	Kr, hkb	18
											Kr, tll	13
1	hbP, Kr, kni	2							hkb	2	Kr, hkb	22
1.1									hkb	4	Kr, hkb	22
1.2	hbP, tll, hkb	216	hbP, tll, hkb	377	hbP, tll, hkb	313	hkb	1	gt, kni	57	Kr, hkb	34
									hkb	6	gt, kni	29
											hkb	1
1.3	hbP, tll, hkb	211	hbP, tll, hkb	337	hbP, tll, hkb	239			gt, kni	33	Kr, kni	116
	hkb	2							hkb	7	Kr, hkb	39
											hkb	2
											gt, kni	13
											hbP, tll hkb	1
1.4	hbP, tll, hkb	197	hbP, tll, hkb	303	hbP, tll, hkb	186	gt, kni	98	gt, kni	15	Kr, kni	90
							hbP, tll, hkb	17	Kr, kni	18	Kr, hkb	42
									hkb	9	hkb	7
											gt, kni	6
1.5	hbP, tll, hkb	189	hbP, tll, hkb	269	hbP, tll, hkb	139	gt, kni	60	gt, kni	4	Kr, kni	65
			Kr, kni	13			Kr, kni	16	Kr, kni	5	Kr, hkb	41
							hbP, tll, hkb	6	hkb	15	hkb	15
							hkb	2			gt, kni	2

Figure S10

A

Number of Gap Gene Combinations Unique to bcd RNAi

Threshold	T = 1	T = 2	T = 3	T = 4	T = 5	T = 6
0.5	2	0	1	1	0	1
0.6	0	0	0	0	0	1
0.7	1	0	0	0	0	2
0.8	1	0	0	0	0	3
0.9	1	0	0	1	1	2
1	0	0	0	1	2	3
1.1	0	0	0	1	0	2
1.2	0	1	0	1	1	2
1.3	0	0	0	1	0	2
1.4	1	0	0	1	1	2
1.5	1	2	0	2	1	2

C

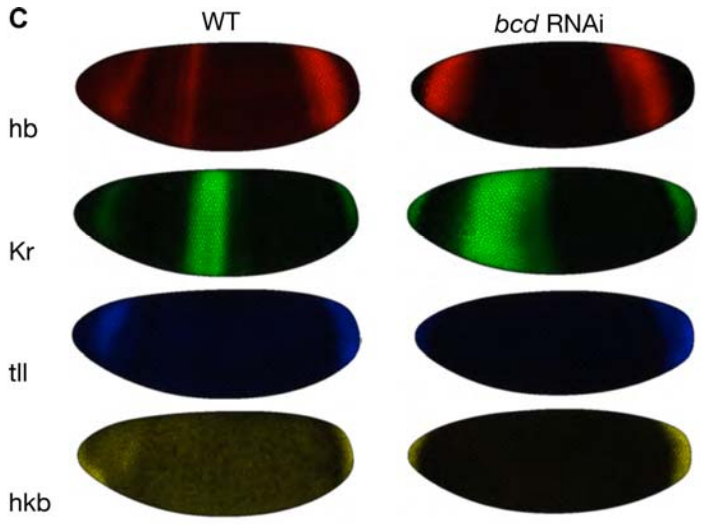

B **Identies and Counts of Gap Gene Combinations Unique to bcd RNAi course atlas**

Threshold	T = 1 Genes	Count	T = 2 Genes	Count	T = 3 Genes	Count	T = 4 Genes	Count	T = 5 Genes	Count	T = 6 Genes	Count
0.5	hb, gt, tll, hkb	13			none	90	**Kr, kni, tll**	**2**			hb, tll, hkb	20
	tll	10										
0.6											hb, tll, hkb	7
0.7	hkb	1									hb, tll, hkb	4
											**hb, Kr, tll**	**1**
0.8	hkb	10									hb, Kr, tll, hkb	3
											hb, tll, hkb	3
											**hb, Kr, tll**	**2**
0.9	hkb	9					hb, tll, hkb	23	hb, hkb	2	hb, tll, hkb	3
											hb, Kr, tll, hkb	1
1							hb, tll, hkb	14	**Kr, tll, hkb**	**2**	Kr, hkb	30
									**hb, hkb**	**1**	hb, Kr, tll	1
											**hb, tll, hkb**	**1**
1.1							hb, tll, hkb	9			Kr, hkb	33
											**hb, tll, hkb**	**1**
1.2			hb, hkb	1			**hb, tll, hkb**	**5**	**hb, tll, hkb**	**1**	Kr, hkb	41
											**hb, tll, hkb**	**1**
1.3							**hb, tll, hkb**	**1**			Kr, kni	85
											Kr, hkb	45
1.4	hb, tll, hkb	216					gt, kni	116	Kr, kni	20	Kr, kni	63
											Kr, hkb	47
1.5	hb, tll, hkb	181	Kr, kni	13			gt, kni	85	Kr, kni	3	Kr, kni	37
			hb, tll, hkb	1			Kr, kni	6			Kr, hkb	48

Figure S11

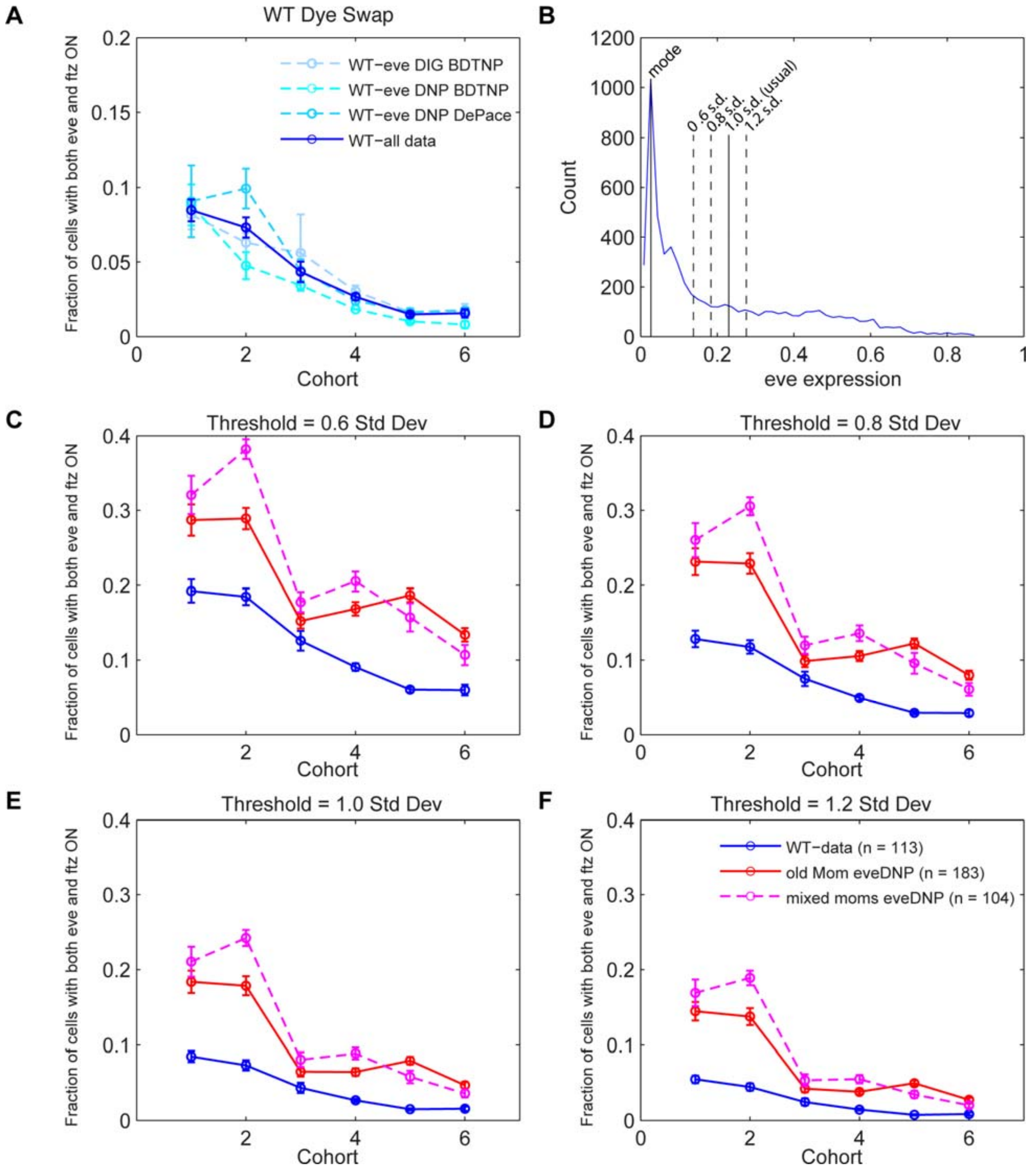
A
WT Dye Swap
WT−eve DIG BDTNP
WT−eve DNP BDTNP
WT−eve DNP DePace
WT−all data
Fraction of cells with both eve and ftz ON
Cohort
B
mode
0.6 s.d.
0.8 s.d.
1.0 s.d. (usual)
1.2 s.d.
Count
eve expression
C
Threshold = 0.6 Std Dev
D
Threshold = 0.8 Std Dev
E
Threshold = 1.0 Std Dev
F
Threshold = 1.2 Std Dev
WT−data (n = 113)
old Mom eveDNP (n = 183)
mixed moms eveDNP (n = 104)

Table S1: Sequences of the enhancer *lacZ* reporter constructs included in the *bcd* RNAi atlas.

Construct	Enhancer sequence	Reference	Primer sequence
**eve1**	AGGCCTAATCACTTCCCTGAAATGCATAATTGTGCCGCGGCTTTTGATACGCTCCTGGCGGAGAGGGAGATGAGGAAA<br>GGATGCACGGGAACCGCAGCCAAGTGGCAGTCGAGATTGGCAAATCCGCCAGCGGACAATGCCCAGAGAATGGGCA<br>ACAAGTAGCGGCGAATTAGCAATCCTATCATGCTTTTATGGCCGGCCAACTCTTGCCCGCGCATCTCAGTTCATCCGAA<br>GCGGGACCAGGTCCAGGTTCAAGTCGAGGTCCAGTACCCCTGCTATCCCGTCAACCCCTTTAGGGCGATAATCCTTCT<br>AAATGTTTGCATTAATTTCGAGGCGTGGACGGATTAGGGCGTGCTGGCTGGGCGGAACCCGCAGCAGAAACCGCCGA<br>GGACACTGCACCGACTGACCTGCAGCCTACAGATCTCTGATCTTCGATCTCTAATCCTTTCGCATTTGCAACTGACTTC<br>TGCACTGGGTCCGCCCCTAATCCTTCCGCCGAGAAGGCGGCAGAGTCGCGAGGTACTGGCCCGGGGTAATGGGATTA<br>TCTGCGATTACCCCAGATGATCCGCAGAAAGTCAATCTGGTTCAGGGGCTAATTGTCAGCGAAGTCAACTAAATCCAAT<br>CCTTTCGCGCCCCCTTCTGTTTATTTGTTTGTTTTCGTTTGTTTTGAGAATTTCTGGCAATTAAGTTGCCCGTTTTGATGC<br>GCGGGGGCGGGTGCATCAAATCCTTTCGGCATACCTGTCCTGCACAAATGCTGAATTCCGCATCCCATGGATACCCAG<br>ATATTCAGATATCCCAAGGC	Fujioka et al., 1999	AGGCCTAATCACTTCCCTG<br>GCCTTGGGATATCTGAATATCTGG
**eve46**	AGGATCCCTGGGCTCTGGGCTCTGGACTATCCGCCGACCCTCCATATCCATGATTTACAATTCTCGTTTTTTTCGCGTTA<br>TTTTTTTAGGGGCTTTAATGACCGTCGTAAAGCCGCAGGAGGACCAGGACCAGGACTCTGCTCACATTTCGCGCACTG<br>ATTCTAAAAAATGAAATCATTTTTTCTTGAATTTCACGGCGCGCCTCGAGCAGGACTCTTTGTTCTCGGCCAGGCAATTG<br>TCCTTTTTTGCGCTCAGCTCTCAGTTTTTTCGTCCAGCGGGCATTACCTACACGGCGTTTTATGGCGGAGATGATATTCG<br>CCTGGGATCGGTTCCGTTTTTTAGGCCATAAAAATTAGGCGGCATAAAAAAACTGCATTGGAATTCTAGTTCTAGTTTCA<br>AGTTTTTAGGTTTCCAGGTTTCTGCCAGCCCGCCTAGATTCGCATTTCGCGGAATTCGGAAGCGGAACAGAATGCCAG<br>AATGGTCAGAATCCTGGCTGACCTTGCCTTTTGGCCAGGGGCCGTAAAAAAATTGACTCGCTGCGGTGCGCGGAATAT<br>TTTTTAAATCTGACTTTCCAACAATCTCTGATCTGGGTTCGAATCGTAAAAAAAAAGCAGAACAAAAAGCGGGCATTTTC<br>GTCGGCAAATGATCTGTTAATGGGCCGGGCTAAAAAACTAAGTCACAAAGTCACAAGGTTGTCCGGTAAATTGACCCG<br>GTTAAGAATGTCTGTCTGTACCGAGAAGGATGCAGGACATTCAGCACTTCAAAGCTCCCACCGCTCGAAGGATTCCCC<br>CGAAGATTCAC	Fujioka et al. 1999	AGGATCCCTGGGCTCTG<br>GTGAATCTTCGGGGGAATCC
**eve5**	ATATCCCAAGGCCGCAAAGTCAACAAGTCGGCAGCAAATTTCCCTTTGTCCGGCGATGTGTTTTTTTTTTAGCCATAACT<br>CGCTGCATTGTTTGGGCCAAGTTTTTCTTCTGCCAAATTGCGGAGATGATGCGGGGATTATGCGCTGATTGCGTGCAAT<br>TATGGACATCCTGCGAGGCCCCGAGGAACTTCCTGCTAAATCCTTTCATCCGCCTACAGAACCCCTTTGTGTCCCGTTC<br>GCCGGGAGTCCTTGACGGGTCCTTCGACTATTCGCTTACAGCAGCTTGCGTAAAATTTCATAACCCTACGAGCGGCTCT<br>TCCGCGGAATCCCTGGCATTATCCTTTTTACCTCTTGCCAATCCGTTGGCTAAAAAACGGCTTCGACTTCCGCGTAACT<br>GCTGGACAACAAAGACAAAAAACGGCGAAAGGACGGCGATTTCCAGGTAGCATTGCGAATTCCGTCAAACTAAAGGAC<br>CGGTTATATAACGGGTTTATATGGCCAGAATCTCTGCATCTCCACGACCGCCAGAAGCTGCGTAAAACTGCAGGCTCTG<br>TTTTGATTTCTGCAACTTCAGTTAATTGCCCGGGATGGCCAGCAATTGCCGGCAATTATAAAACAGCGCAGATGTGACT<br>CAGCTTCCATATCTAACTCTATATCTCATGCCGAAAATCTAGGGTGGGGAGCGGAGGGGCGGGGTGCGTGGGTGACTT<br>GCCTGCCAGGGAAAGGGGGCGGGGGTTCAGCGGGTGATAAATGTGCGTGATTTGGAATGAATGCGCATCGATTAAAA<br>CCGCAGGGCAATCAATTT	Fujioka et al. 1999	ATATCCCAAGGCCGCAAAG<br>AAATTGATTGCCCTGCGGT
**eve3+7**	GGATCCTCGAAATCGAGAGCGACCTCGCTGCATTAGAAAACTAGATCAGTTTTTTGTTTTGGCCGACCGATTTTTGTGC<br>CCGGTGCTCTCTTTACGGTTTATGGCCGCGTTCCCATTTCCCAGCTTCTTTGTTCCGGGCTCAGAAATCTGTATGGAATT<br>ATGGTATATGCAGATTTTTATGGGTCCCGGCGATCCGGTTCGCGGAACGGGAGTGTCCTGCCGCGAGAGGTCCTCGC<br>CGGCGATCCTTGTCGCCCGTATTAGGAAAGTAGATCACGTTTTTTGTTCCCATTGTGCGCTTTTTTCGCTGCGCTAGTTT<br>TTTTCCCCGAACCCAGCGAACTGCTCTAATTTTTTAATTCTTCACGGCTTTTCATTGGGCTCCTGGAAAAACGCGGACA<br>AGGTTATAACGCTCTACTTACCTGCAATTGTGGCCATAACTCGCACTGCTCTCGTTTTTAAGATCCGTTTGTTTGTGTTTG<br>TTTGTCCGCGATGGCATTCACGTTTTTACGAGCTC	Small et al., 1996	GGATCCTCGAAATCGAGAGC<br>GAGCTCGTAAAAACGTGAATGC
**eve2**	GGTTACCCGGTACTGCATAACAATGGAACCCGAACCGTAACTGGGACAGATCGAAAAGCTGGCCTGGTTTCTCGCTGT<br>GTGTGCCGTGTTAATCCGTTTGCCATCAGCGAGATTATTAGTCAATTGCAGTTGCAGCGTTTCGCTTTCGTCCTCGTTTC<br>ACTTTCGAGTTAGACTTTATTGCAGCATCTTGAACAATCGTCGCAGTTTGGTAACACGCTGTGCCATACTTTCATTTAGA<br>CGGAATCGAGGGACCCTGGACTATAATCGCACAACGAGACCGGGTTGCGAAGTCAGGGCATTCCGCCGATCTAGCCA<br>TCGCCATCTTCTGCGGGCGTTTGTTTGTTTGTTTGCTGGGATTAGCCAAGGGCTTGACTTGGAATCCAATCCCGATCCC<br>TAGCCCGATCCCAATCCCAATCCCAATCCCTTGTCCTTTTCATTAGAAAGTCATAAAAACACATAATAATGATGTCGAAGG<br>GATTAGGGG	Small et al., 1991	GGTTACCCGGTACTGCATAAC<br>CCCCTAATCCCTTCGACATC
**eve46mini**	TCGAGCAGGACTCTTTGTTCTCGGCCAGGCAATTGTCCTTTTTTGCGCTCAGCTCTCAGTTTTTTCGTCCAGCGGGCAT<br>TACCTACACGGCGTTTTATGGCGGAGATGATATTCGCCTGGGATCGGTTCCGTTTTTTAGGCCATAAAAATTAGGCGGCA<br>TAAAAAAACTGCATTGGAATTCTAGTTCTAGTTTCAAGTTTTTAGGTTTCCAGGTTTCTGCCAGCCCGCCTAGATTCGCA<br>TTTCGCGGAATTCGGAAGCGGAACAGAATGCCAGAATGGTCAGAATCCTGGCTGACCTTGCCTTTTGGCCAGGGGCC<br>GTAAAAAAATTGACTCGCTGCGGTGCGCGGAATATTTTTTAAATCTGACTTTCCAACAATCTCTGATCTGGGTT	Fujioka et al., 1999	TCGAGCAGGACTCTTTGTTCTC<br>AACCCAGATCAGAGATTGTTGG
**hb posterior**	TAGCACGAAAAACCGAAGGATTAAAAAAGGAAACTAGAGCAGAGGTCCCGGGGCAGGGCGAATAGTTGCTCTAATTTTCAT<br>TGTCCGCCTTAATGGTTACGCCGTAAAATTGGCTATGCGGCCAAACAATAGTGCGAAGGACGACGGCAGGACGCGCAGGA<br>CAATCGTCTGGTGGATTTCCAGTCGACACGCCACGAGATTTTATGAAGGCAACTCGCTTTGCATGTTATTCCATAGATTTCG<br>CTTCGGTCCCGGTTTGTTTTGGTCAGGTAAGACCTTCGATTAACAATGAAAGTAGCTGGAAAATCGCGAGAAACTTCGAAA<br>GACACACAAAGATACAATATCTATGAGTCTAATGGTCATTAGAGCGGTGCGCTCTACATACAATTGTACCAGCCGTCTTGTTT<br>GAAGCCTAAAAAACGTCGCAAAAAACACACTTCCGCGTAAGACATCCCATTTCTGTGGTCCGATCGTAAAATATTTAGTTTTT<br>TATGACCAACGGTGCGGGCAGGTAGCTGGCTGCCGTTTTTTGTGCGCGACCTCAACCCTTTCACCCATTAAGAAAAAATC<br>GCATCCTGTGAGTGTCCTTGCCCGTTTCCCTCGAAACGGCCCACAATTTGTGTGCTTTGCGTTTTCTCCTCTCTTTTTGTTT<br>CCACCTAATGTCGGCGTCATTGTCTTCTTTATGACGCCTCGGTTGTTTCTTTTTTATGGTGTCCTTTGTCCTTTGAGCCTCGT<br>TGCACGGCCAAATCCCTACTTCCTCAACCCTTTGGCGGACGAGAAAGTTGCTAGGAGGAGAACGGGTTAAGCGAAAACTC<br>CATTGCACTTTTTACAAGCCGCGATCTTCTTGGAATTAGTTTTGGTCATTAGGCGAAAGGGTTAATTTCGATTTTGGCTCTCG<br>GTGGGTTTACTGAGTGAATTCAATGGGCTAAGGCGAGTAAAGGGTTATACTGTTTTTACATTTTACTACTTGGAAAATACTGAA<br>GAACTTGTAGGAAAAATTTCCAGCACTTTTAAAAGCCATATATAACTTTATGAATATGAACTTCAAATGTAAAAACCTGAAAGTG<br>ACATGTAGTTATTTTAAGGTCCTTGAAAATGATCATCGTCTAAAATTTCTTTTTTTTAAATAATTTTTAAAATATTTTTTTGATAGCAT<br>ACGAAGTATTTAAAAATGTGAACAGATTAAACACATTAAATTTATAAAAGTAAATACAACAGATTTAGCATAGAAATAAAAATCATT<br>TTAATGTTCCGTCCATAAGTAACGGTCGTGGAAAATTCTTGAAAATCCCACAAATTATATTCGATCCCTTTGGCCGAACATTTG<br>GTGCGATTACATTCGTAATTCGCTGGAAATTAAGGCCACTAAGTCGCCAGCGAAATGAATTCGGACATTGGGCATTGGACAA<br>ATGTAAAAAGGACTCTAGAGCCCCGACCATTGCAATGGTCCATTGTTGAGCGTCCGAAAGATCTGAAAACCAAACCCAAAC<br>CAAATCCCGAGCTTAGGCAATCGGCATTGGGAAATAAGCGCCAAATATTCTACCCCCCACTCCAAAAACGAGCATT	Wunderlich et al., 2012	

Construct	Enhancer sequence	Reference	Primer sequence
**gt posterior**	AATTTATTACCAGAAACTTACCATCACTTCGAGATGAAAGTGCGGAGGAGAGCGCTGATCTTTGCTTATCCCAAACTAGG<br>GGTTTTTTAGGGGTAAAAAGCGGGAATACAGATTCTCAGAAACAAAATGGCAGTCCTAATATGTGATAAGTTGCTTTCAA<br>CTTACGTTTACGACAGGGGCCATTCGAAGCTAAGGATTCCAATATGCGACTGTTAACCCATTAAGACAAAGGGCCGCGA<br>AAGGAGTTAACGGCAATTCCCCGAAACCAAGCACGAATCCAAATCCAAACCACTCCTCACCCTTTTTTGGACGGGTGG<br>GACGGGTCATATAAGGCAAAACCCCTTTCAGTCAATTAGTCGCTGATTTTCTTTGTCACCTAGCAGCGGACCAATATAAA<br>AATCGCAGCCACAATGGTCGGAGGAGAGAACCCTTTTTTTTTTAAGGACCGCCGGTGTCCGAAATATCAGTTTATGGCT<br>CCTTAAAAAACTGCAGCGGTTTTAGGGCCCGCGGACTCGGAATGAGGCCTTTTCGCACGAAGCGTCCATTATGCAATA<br>AAACTTTGGATGTTTTTTAGGCAACAGCATAACAGCCTAATACAGCGCATACCGTGGGTGGGCAACCCGTTCGGCCATC<br>AGGTAGATGATTCCGTAAAAAACGCGCCCAAGGTCACAACTCAAAGGATTGCACAAATATTGCATCTTTGGTTCGGAGC<br>TCATTATGGCGAAGGAACAGCAGTCCGAGTATATGAATATATGGTTTTATGGCCTGCTGTTTTTTACGTTTTTTTTTCGCT<br>GAATCTGGTTTTTACACCGAACTTGGCAACTCTTTGGCTTCGACTTCGGCCGCGACTTTTTATGGCATCTGCTCGGGAT<br>CGATTGGGCTGGGCTATACGTATGTCCAGGATCTGGAAGGGCTCGGGTTCGGGCCCGGTAACCGCAGGTAAGGGATC<br>GACTCAATGACGGCGACGTGACGGTCCGAGTTTTAGTTTAATCCACCATTTTTACGGTTGGATTAGCGCACGGATTAGC<br>GGATAAGTTCGCGGTTTTTTACTGATTACCATCGATCGATCCCTCTTTTTATTTGCAATTCACCCAATCCCCCTGAATGGG<br>CAGTTGTAAA	Wunderlich et al., 2013	
**eve late seven stripe**	-6.4kb (NdeI) to -4.8kb	Fujioka et al., 1996	-6.4kb (NdeI) to -4.8kb
**eve whole locus reporter**	Begins -6.4 kb upstream of *eve* transcription start site (TSS) and ends 11.3 kb downstream of *eve* TSS. The *eve* coding sequence has been replaced with *LacZ* and the neighboring *TER94* gene has been fused to GFP	Gift from M. Fujioka	

Table S2: The numbers of individual embryos for each gene at each time point included in the *bcd* RNAi gene expression atlas

Genes	T=1	T=2	T=3	T=4	T=5	T=6
D	9	19	26	13	5	13
Kr	10	13	16	10	8	10
hb posterior enhancer	6	8	20	9	6	3
gt posterior enhancer	21	18	29	13	6	3
eve3+7 enhancer	3	10	13	7	5	10
eve4+6 enhancer	10	27	26	16	8	8
eve5 enhancer	6	16	32	12	6	9
eve4+6mini enhancer	3	8	12	11	0	3
eveLocus lacZ	6	20	13	13	4	2
cad	5	15	24	14	3	6
eve	17	22	54	34	32	23
fkh	5	14	16	9	4	7
ftz	38	77	94	30	19	32
gt	25	30	31	13	8	9
h	8	11	11	9	14	15
hb posterior enhancer	16	12	13	17	10	12
hkb	16	11	20	14	6	8
kni	9	15	14	8	6	8
run	8	20	22	16	9	5
tll	15	20	20	11	6	6
Hb protein	7	10	14	7	6	9
Average	11.6	18.9	24.8	13.6	8.1	9.6
Sum	243	396	520	286	171	201
Total	1817					

Table S3: The standard deviations of each gene in the *bcd* RNAi gene expression atlas. Data from the WT atlas is included for comparison (Fowlkes et al., 2008).

Gene Name	WT s.d.	bcd RNAi s.d.
cad	0.165	0.067
eve	0.129	0.143
fkh	0.068	0.029
ftz	0.131	0.175
gt	0.108	0.073
hb	0.134	0.059
hkb	0.106	0.037
kni	0.099	0.061
Kr	0.066	0.079
tll	0.063	0.036
D	0.108	0.103
h	0.167	0.163
run		0.154
hbProtein	0.132	0.108
hb posterior enhancer		0.063
gt posterior enhancer		0.078
eve3+7 enhancer		0.075
eve4+6 enhancer		0.089
eve5 enhancer		0.131
eve4+6mini enhancer		0.077
eve locus reporter		0.104

Table S4: ON/OFF thresholds used in the combination analysis in Figs 5, S8, S9.

**WT**						
	**T = 1**	**T = 2**	**T = 3**	**T = 4**	**T =5**	**T =6**
**gt**	0.35	0.36	0.34	0.27	0.24	0.21
**Kr**	0.34	0.32	0.28	0.23	0.21	0.20
**kni**	0.32	0.30	0.31	0.34	0.35	0.32
**tll**	0.29	0.24	0.19	0.18	0.17	0.16
**hkb**	0.24	0.30	0.29	0.27	0.23	0.21
**hb mRNA**	0.41	0.28	0.23	0.23	0.28	0.25
**hb Protein**	0.21	0.20	0.22	0.22	0.22	0.16
**bcd RNAi**						
**gt**	0.27	0.30	0.31	0.26	0.22	0.17
**Kr**	0.24	0.31	0.31	0.32	0.26	0.24
**kni**	0.20	0.23	0.27	0.29	0.25	0.20
**tll**	0.26	0.28	0.24	0.19	0.15	0.10
**hkb**	0.15	0.21	0.24	0.26	0.19	0.18
**hb mRNA**	0.21	0.26	0.28	0.25	0.22	0.23
**hb Protein**	0.08	0.13	0.14	0.24	0.27	0.35
